# Topological N-glycosylation and site-specific N-glycan sulfation of influenza proteins in the highly expressed H1N1 candidate vaccines

**DOI:** 10.1038/s41598-017-10714-2

**Published:** 2017-08-31

**Authors:** Yi-Min She, Aaron Farnsworth, Xuguang Li, Terry D. Cyr

**Affiliations:** Centre for Biologics Evaluation, Biologics and Genetic Therapies Directorate, Health Canada, Ottawa, Ontario K1A 0K9 Canada

## Abstract

The outbreak of a pandemic influenza H1N1 in 2009 required the rapid generation of high-yielding vaccines against the A/California/7/2009 virus, which were achieved by either addition or deletion of a glycosylation site in the influenza proteins hemagglutinin and neuraminidase. In this report, we have systematically evaluated the glycan composition, structural distribution and topology of glycosylation for two high-yield candidate reassortant vaccines (NIBRG-121xp and NYMC-X181A) by combining various enzymatic digestions with high performance liquid chromatography and multiple-stage mass spectrometry. Proteomic data analyses of the full-length protein sequences determined 9 N-glycosylation sites of hemagglutinin, and defined 6 N-glycosylation sites and the glycan structures of low abundance neuraminidase, which were occupied by high-mannose, hybrid and complex-type N-glycans. A total of ~300 glycopeptides were analyzed and manually validated by tandem mass spectrometry. The specific N-glycan structure and topological location of these N-glycans are highly correlated to the spatial protein structure and the residential ligand binding. Interestingly, sulfation, fucosylation and bisecting N-acetylglucosamine of N-glycans were also reliably identified at the specific glycosylation sites of the two influenza proteins that may serve a crucial role in regulating the protein structure and increasing the protein abundance of the influenza virus reassortants.

## Introduction

Influenza viruses are among the most virulent pathogens, causing severe respiratory illness and human pandemic deaths worldwide. Frequent mutations of viral proteins (“antigentic drift”) and the occasional presence of novel subtypes (“antigenic shift”) of influenza A viruses result in the emergence of unpredictable virus strains, rendering the prevention of influenza epidemics or pandemics difficult. Rapid understanding of influenza viruses is highly beneficial to the timely generation of high-yield candidate vaccines and the most cost-effective vaccination programs. In past years, several candidate reassortant vaccines such as NIBRG-121xp and NYMC-X181A derived from A/California/07/2009 pandemic influenza viruses have been developed through serial egg passages of viruses derived from either conventional recombination techniques used by Dr. Bucher’s laboratory at the New York Medical College (NYMC) or a reverse genetics technology system by the National Institute for Biological Standards and Control (NIBSC, UK)^1–3^. The strategy to improve the growth of human influenza viruses utilized single amino acid mutations in the two major surface membrane glycoproteins of hemagglutinin (HA) and neuraminidase (NA), which create an additional glycosylation site at the structural motif Asn-X-Ser/Thr/Cys (to lesser extent at Cys, X is any amino acid except proline) or remove an existing glycosylation site of the proteins. Studies have demonstrated a significant increase in the expression level of viral proteins by these mutated influenza viruses, relative to their parent strains without altering antigencity^2, 3^.

N-glycosylation of influenza proteins dynamically regulates protein folding, stability, solubility, protein biosynthesis, enzymatic activity and antigenicity. N-glycans attached to asparagine residues of proteins are usually localized at the sequence motif of Asn-X-Ser/Thr/Cys. However, the presence of such motifs is often not sufficient to generate N-glycosylation at a given site, which is also dependent on the flanking structural surface topology and close interaction with the processing enzymes and glycosyltransferases^4^. N-glycan structures are established on a trimannosyl core of Man_3_GlcNAc_2_ (Man: mannose; GlcNAc: N-acetylglucosamine), and its branch extension to other monosaccharides including Man, Gal (galactose), GlcNAc, Fuc (fucose) and Sial (sialic acids) of high mannose-, complex- and hybrid-type glycans. Most interesting of these are the complex glycans of influenza glycoproteins usually featuring a large diversity of bi-, tri- and tetra-antennary structures with additional modifications of fucosylated, sialylated, sulfated groups on the carbohydrate side chains. However, the physiological functions associated with those distinct complex N-glycan structures and specific locations are still largely unknown.

To thoroughly examine the glycosylation states and the structural consequences caused by mutations of HA and NA in the high-yielding pandemic H1N1 candidate reassortant vaccines, we utilized high-resolution LTQ-FT and Orbitrap mass spectrometry-based proteomics approaches to identify the N-glycan structure of intact glycopeptides of the protein digests by low-energy collision-induced dissociation (CID) followed by multi-stage tandem mass spectrometry (MS^3^). In previous studies, we quantitatively analyzed the candidate vaccines of NIBRG-121xp and NYMC-X179A both derived from the influenza virus strain A/California/7/2009 by different manufacturers, and evaluated the antigenic stability of the HA and NA proteins^3, 5^. We subsequently identified the surface modifications of influenza proteins induced through virus inactivation by *beta*-propiolactone which have little impact on the HA antigenicity and NA enzyme activity^6^. In terms of the large diversity of glycan structures, the heterogeneity of glycan-protein conjugates of proteins HA and NA modulate and influence a variety of protein functions such as receptor binding, enzymatic activity and antigenicity^7–19^. Despite the considerable progress in the determination of 3D crystal structures and post-translational modifications of influenza proteins^20–30^, much remains to be understood about the structure-function relationship between the specific compositions of N-glycans and the biosynthesis of influenza viruses.

## Results

### Structural identification of influenza glycopeptides using liquid chromatography and multiple-stage mass spectrometry

Sequence alignment of influenza proteins derived from two candidate vaccines of NIBRG-121xp and NYMC-X181 revealed the amino acid identities of 99.12% in HA and 99.79% in NA (Supplementary Information Figs S1 and S2). These surface membrane proteins were predicted to share 11 and 7 structural Asn-X-Ser/Thr/Cys glycosylation motifs of HA and NA derived from the two candidate vaccines (Supplementary Information Fig. S3), respectively. The unique glycosylation site was located on either Lys136Asn of HA (NIBRG-121xp) or Asn88Gly of NA (NYMC-X181), which was demonstrated to have a significant effect on the growth yields of influenza viruses^2^.

**Figure 1:**
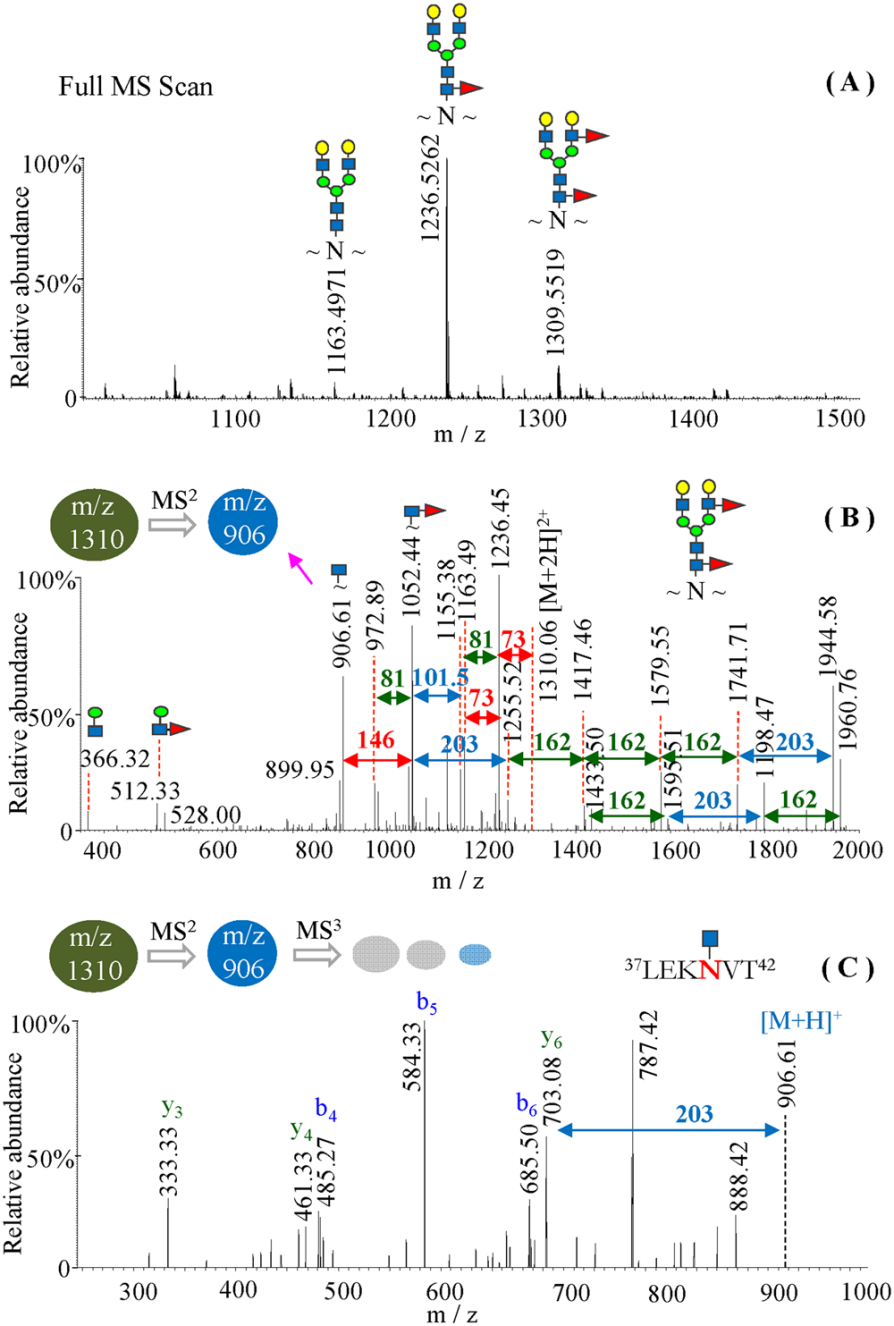
Multiple-stage MS/MS analysis of a hemagglutinin glycopeptide of the doubly charged ion of m/z 1309.5519 by LTQ-FT mass spectrometry. (**A**) Full MS scan of the PGC fraction at 25% acetonitrile elution of a tryptic digest followed by protease K cleavage. (**B**) Glycan sequencing by MS/MS at the precursor ion of m/z 1309.55. (**C**) Peptide sequencing by MS^3^ fragmentation of the abundant monoglycosylated peptide ion at m/z 906.61. Yellow circle, galactose; blue square, N-acetylglucosamine; green circle, mannose; red triangle, fucose.

Our previous study using LC MS/MS analyses has shown that a delipidation of the protein materials followed by various enzymatic digestions was required to achieve the complete sequence coverage of HA^6^. Using the same procedure and porous graphitic carbon (PGC) affinity purification of glycopeptides, we have carried out a thorough examination of N-glycan structures of the influenza proteins in the vaccines of NIBRG-121xp and NYMC-X181. PGC columns have been widely used for the purification, separation and selective enrichments of glycans or glycopeptides through either online LC-MS or offline solid phase extraction with PGC cartridges^31–34^, LC/MS/MS analyses of the influenza glycopeptides purified by PGC affinity column in our experiments are thus not significantly biased regarding the type of glycan structural identification. Following a single MS scan, within the same LC/MS/MS run monoglycosylated peptide ions with only N-acetylglucosamine were then selected and further fragmented by MS^3^ to generate specific peptide fragments for reliable amino acid sequence determination. Figure 1 showed the typical identification of both the glycan structure and the peptide sequence of a HA glycopeptide at the doubly charged ion of m/z 1309.5519 (Fig. 1A). MS/MS spectrum of the precursor ion at m/z 1309.56 (Fig. 1B) revealed two diagnostic glycan ions of m/z 366.2 (HexHexNAc^+^) and m/z 528.2 (HexHexHexNAc^+^), and the specific fragments resulting from sequential losses of neutral saccharides of galactose, N-acetylglucosamine, fucose and mannose subunits in the glycan structure. Whereas, the glycopeptide sequence was reliably defined by the N-terminal b_m_ (m = 4–6) fragment ions and the C-terminal y_n_ (n = 3–6) ions from the MS^3^ collision-induced dissociation of the MS/MS daughter ion of m/z 906.61 (Fig. 1C). Based on the identity of the peptide sequence of HA at residues 37–42 and the complex N-glycan attachment, the theoretical mass of the intact glycopeptide was then calculated as 2618.0959 Da which is closely matched to the measured value of 2618.0960 Da. The highly accurate mass measurement using LTQ-FT mass spectrometry thus further validated the MS/MS sequencing results of the glycopeptide.

Additional modification, caused by *in vitro* chemical reactions, might render a complex interpretation of MS/MS spectra of the influenza glycopeptides. As reported previously^6^, *beta*-propiolactone, as the virus inactivation reagent employed, ubiquitously reacted with several electron-pair-donor amino acid residues at the surface of the influenza proteins with a reactivity decreasing in the order of Cys > Met > His > Asp/Glu > Tyr > Lys > Ser > Thr. Although the steric hindrance of large glycan chains prevents the chemical reactions with *beta*-propiolactone at nearby amino acid residues, one tryptic glycopeptide at residues 301–322 (peptide sequence: GAINTSLPFQNIHPITIGKCPK; retention time: 37.50 min) of HA was found to be partially modified at Lys319 accompanied by additional masses of ~72 Da on a set of peaks (retention time: 35.34 min) which matched well to the theoretical mass of *beta*-propiolactone (72.0211 Da) (Supplementary Information Fig. S4). This *beta*-propiolactone modification specifically occurred on the HA peptide 301–322 with the high-mannose glycans (Man_5_-Man_8_), but not other glycopeptides containing hybrid or complex N-glycans.

Cys-carbamidomethylation is a deliberate modification introduced by DTT reduction and alkylation by iodoacetamide during sample preparation. Manual inspection of MS/MS product ion spectra also displayed the presence of S-carbamidomethylation at methionine with a characteristic neutral loss of 105 Da from the glycopeptide 488–497 of HA (CDNTCMESVK) (Supplementary Information Fig. S5). The single mass spectrum of the triply charged ion at m/z 839.98 showed the selective carbamidomethylation of Cys488, Cys492 and Met493, and the MS/MS spectrum yielded abundant losses of 52.8 units on the doubly charged fragments and 35.2 units on the triply charged fragment ions, corresponding to a molecule of 2-(methylthio)acetamide (C_3_H_7_NOS, 105.0248 Da). Similar to those reported previously^35, 36^ and the recent analysis of influenza H5N7 hemagglutinin^37^, the occurrence of such atypical fragments highlights the need of careful data interpretation of glycopeptides in the sequence analysis of influenza proteins.

### Site-specific N-glycan sulfation of the influenza proteins

**Figure 2:**
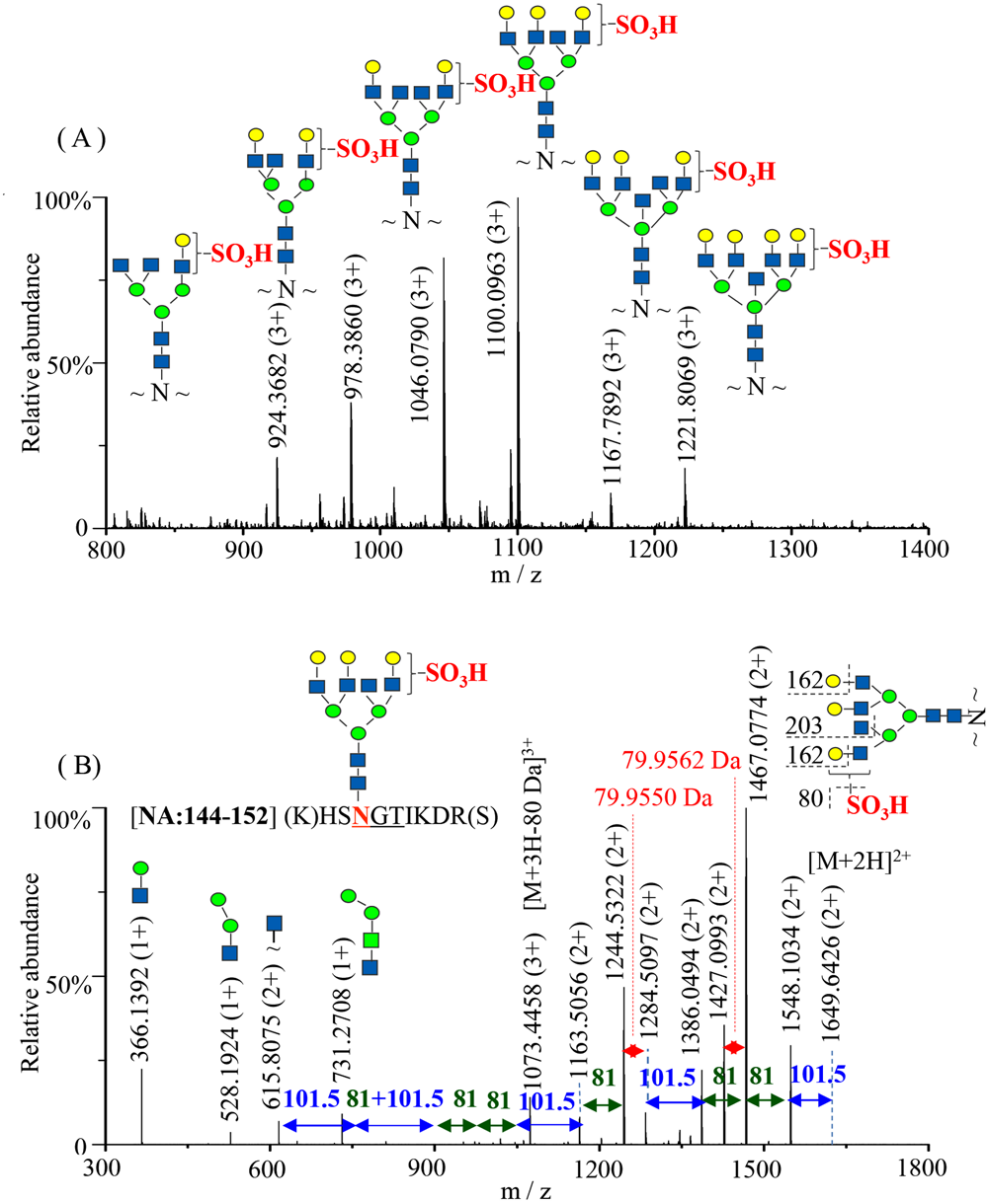
The structural N-glycan analyses of the tryptic neuraminidase peptide 144–152 isolated from the influenza vaccine NIBRG-121xp by LC MS/MS with high-resolution Orbitrap Fusion mass spectrometry. (**A**) MS spectrum of the peptide profile containing sulfated and bisected GlcNAc complex-type N-glycan forms. (**B**) MS/MS spectrum of the triply charged ion at m/z 1100.0963 (3 + ). The low energy collision-induced dissociation (CID) of the precursor ion displayed not only the consecutive losses of neutral saccharides from the glycan structure, as well as a chemical group loss at either 79.9550 Da or 79.9562 Da between the two pairs of doubly charged fragment ions. The accurate mass measurements identified the element composition of 79.9556 ± 0.0006 Da as being derived from a sulfate group (SO_3_, calculated mass: 79.9568 Da), and MS/MS analysis defined the site of monosulfate group at the terminal residue of either Gal or GlcNAc (or the isobaric GalNAc) on the branched N-glycan side chain.

Analyses of various enzymatic digests of the influenza vaccines identified those glycopeptides that have common N-glycan compositions of high-mannose, hybrid and complex N-glycans on the structural sequence motif Asn-X-Ser/Thr/Cys of HA and NA. Unexpectedly, a group of glycopeptide peaks from the tryptic digest of the influenza vaccine NIBRG-121xp were observed at m/z 1046.0804, m/z 1100.0983, m/z 1167.7905 and m/z 1221.8086 separating by 162.0537 Da, 203.0766 Da and 162.0543 Da, respectively, but displayed abnormal masses that did not match to any predicted peptide plus a regular N-glycan molecule (Supplementary Information Fig. S6A). MS/MS spectrum of the triply charged ion at m/z 1100.0983 revealed a neutral loss of 80 Da following the sequential losses of two molecules of galactose (162 Da) and N-acetylglucosamine (203 Da) (Supplementary Information Fig. S6B). The MS^3^ fragmentation of the relatively high-abundance glycopeptide ion with N-acetylglucosamine confirmed the peptide sequence at residues 144–152 of NA was not modified on the peptide backbone except the Asn residue. Considering the fragmentation pattern of neutral losses of saccharides on the MS/MS spectrum of the glycopeptide, the additional modification of 80 Da could be inferred to be located on the terminal residue of either Gal or GlcNAc (or the isobaric GalNAc) of the glycan side chain. Compared to the measured value of m/z 3298.2793 (MH^+^), accurate mass calculation of glycopeptide 144–152 defined the 80 Da modification as the incorporation of a sulfate group (MH^+^, 3298.2779), rather than a phosphate group due to a higher mass error from its calculated mass of m/z 3298.2874. The location of a sulfate group at the second position of GlcNAc (or GalNAc) is more likely based on the previous reports on influenza proteins using biochemical characterization of the HA receptor glycan binding^38^, 3D structure of NA^39, 40^ and bioinformatics analysis of sulfated glycan structures^41^. Further data interpretation of the adjacent glycopeptide peaks identified a series of sulfated glycans together with two bisecting GlcNAc structures as illustrated in Fig. S6, although the other structural isomers such as penta-antennary complex glycans could not be ruled out by the simple MS/MS fragmentation^42, 43^. Subsequent analysis of glycopeptide 140–150 of NA generated from the tryptic digestion followed by chymotrypsin confirmed the presence of sulfated glycan structures at site Asn146 of NA (Supplementary Information Fig. S7). With the recently technical improvements of the Orbitrap Fusion, we also conducted a retrospective examination on the neutral loss group of 80 Da by high-resolution MS/MS analysis. As expected, Orbitrap CID fragmentations of the precursor ion at m/z 1100.0963 displayed a similar profile to that of the MS/MS spectrum obtained by LTQ-FT ion trap. Figure 2 shows the sequential losses of neutral saccharides from the glycan structure, but also a chemical group loss at either 79.95550 Da or 79.9562 Da between the two pairs of doubly charged fragment ions. The accurate mass measurements thus identified the element composition of 79.9556 ± 0.0006 Da is derived from a sulfated group (SO_3_, 79.9568 Da) instead of a phosphorylated group (PO_3_H, 79.9663 Da) at the branched N-glycan side chain. The structural pattern of sulfated N-glycans of NA in the influenza vaccine NYMC-X181A was identical to those in the influenza vaccine NIBRG-121xp at the highly conserved sequence region at residue Asn146.

Similar analyses by high accuracy mass measurements on the glycopeptide 131–138 of HA, produced from a digest of the influenza vaccine NIBRG-121xp with trypsin followed by proteinase K (Supplementary Information Fig. S8 and Table S3), identified a broad range of sulfated tri- and tetra-antennary glycans at residue Asn136. Interestingly, those sulfated N-glycans were also observed to be highly fucosylated, which were localized at the unique site of Asn136 but absent from that of the influenza vaccine NYMC-X181A.

### N-glycan distribution of influenza proteins

**Figure 3:**
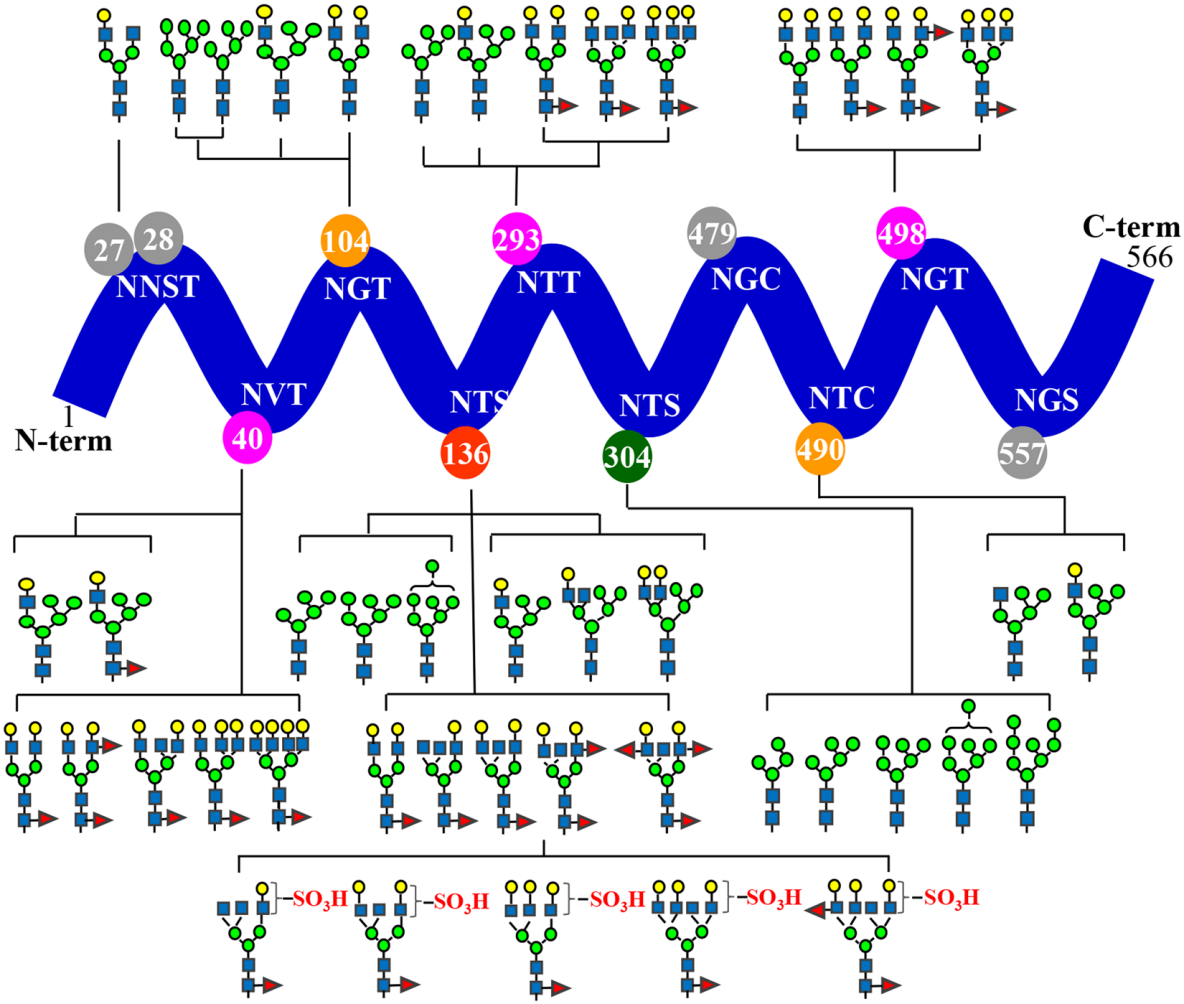
Overview of the site-specific N-glycan distribution of hemagglutinin from the candidate vaccine of NIBRG-121xp. The N-glycosylation microheterogenetity at the individual sites of the protein sequence chain is shown.

**Figure 4:**
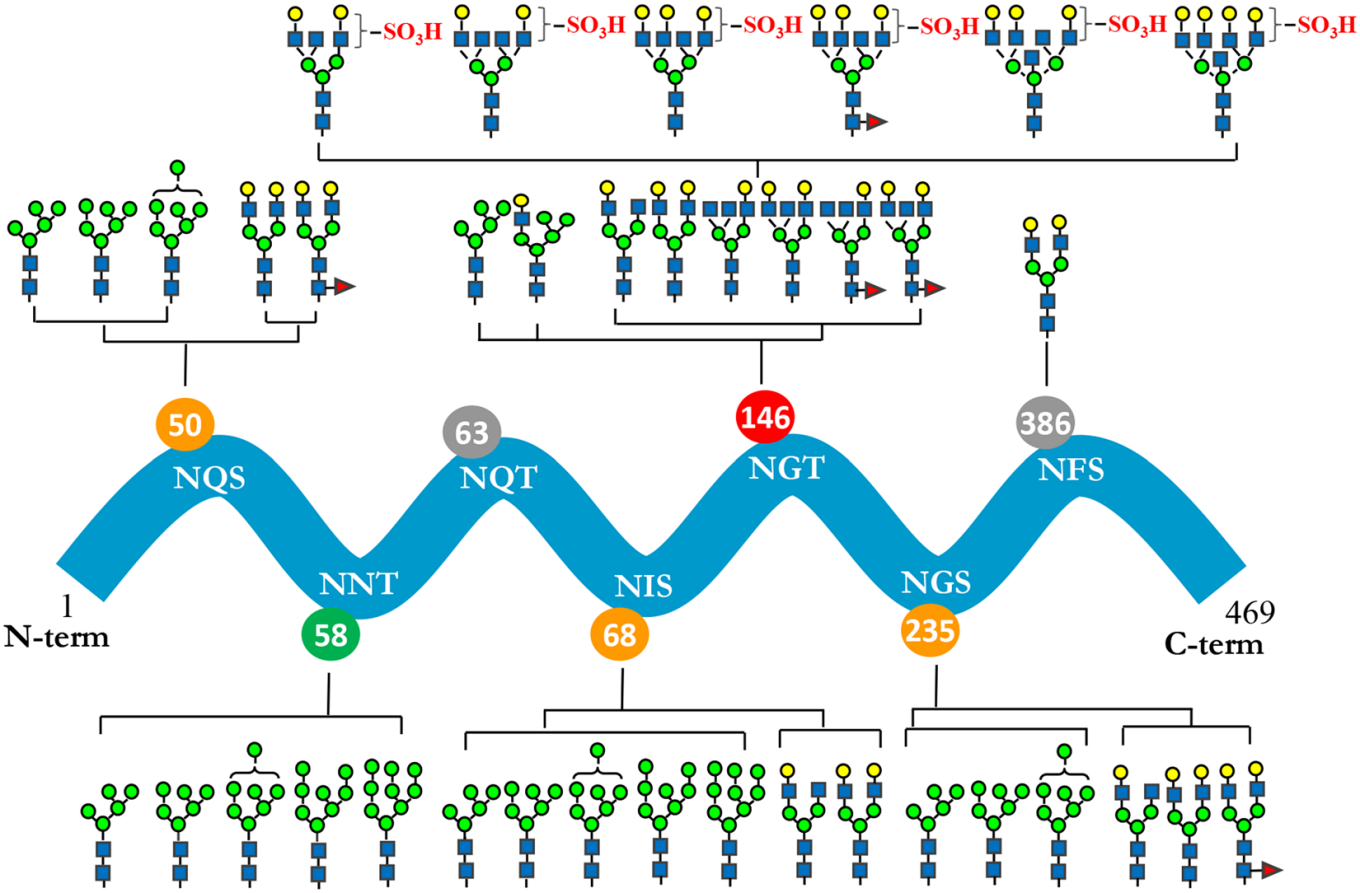
Overview of the site-specific N-glycan distribution of neuraminidase from the candidate vaccine of NIBRG-121xp. The N-glycosylation microheterogenetity at the individual sites of the protein sequence chain is shown.

Mass spectrometric analyses revealed similar N-glycan compositions and profiles of HA and NA at the conserved sequence regions of the respective proteins in the vaccines of NIBRG-121xp and NYMC-X181A except at the specified mutation sites. A detailed list of the detected glycopeptides from various enzymatic digests is summarized in Supplementary Information Tables S1–S5. Figures 3 and 4 display an overall view of the N-glycan structures on the glycosylation sites of HA and NA of the influenza vaccine NIBRG-121xp, respectively.

In HA, 9 N-glycosylation sites were identified as having different types of glycans (Fig. 3). The simplest glycan composition was found at the site of Asn27 or Asn28 near the protein N-terminus to have a single complex glycan of GalGlcNAc_2_Man_3_GlcNAc_2_. In addition, a group of high-mannose N-glycans of Man_4–8_GlcNAc_2_ were observed only at the site Asn304. Fucosylated complex N-glycans were present at four sites of Asn40, Asn136, Asn293 and Asn498. The mutation site of Asn136 of NIBRG-121xp was occupied by an extensive range of N-glycans including high-mannose, hybrid, complex, sulfated and fucosylated glycan structures. The unusual structures of two tetra-antennary hybrid-type N-glycans at site Asn136 in Fig. 3 were assigned by the monosaccharide compositions (Hex_6–7_HexNAc_4,_ Supplementary Information Tables S1 and S3 and S5), MS and MS/MS measurements of the glycopeptides, together with further consideration of the putative egg-derived N-glycan structures reported previously^44, 45^.

The N-glycan libraries at the 6 occupied consensus sites of NA have less complex antennas than those of HA (Fig. 4). A single complex-type glycan of Gal_2_GlcNAc_2_Man_3_GlcNAc_2_ was also found at site Asn386 near the C-terminus of the protein. Likewise, only Man_5–9_GlcNAc_2_ high-mannose type N-glycans were present at position Asn58. Mixed glycoforms of high-mannose and complex N-glycans were found at three glycosylation sites of Asn50, Asn68 and Asn235. Interestingly, like the mutant site of HA, the unique glycosylation site of NA at Asn146 contains a wide variety of highly modified glycans which included a number of high-mannose, hybrid, complex, sulfated and fucosylated N-glycans, together with the rare bisecting N-acetylglucosamine on the core structure of tetra-antennary N-glycan branches.

### Structural topology of N-glycans in the influenza proteins

**Figure 5:**
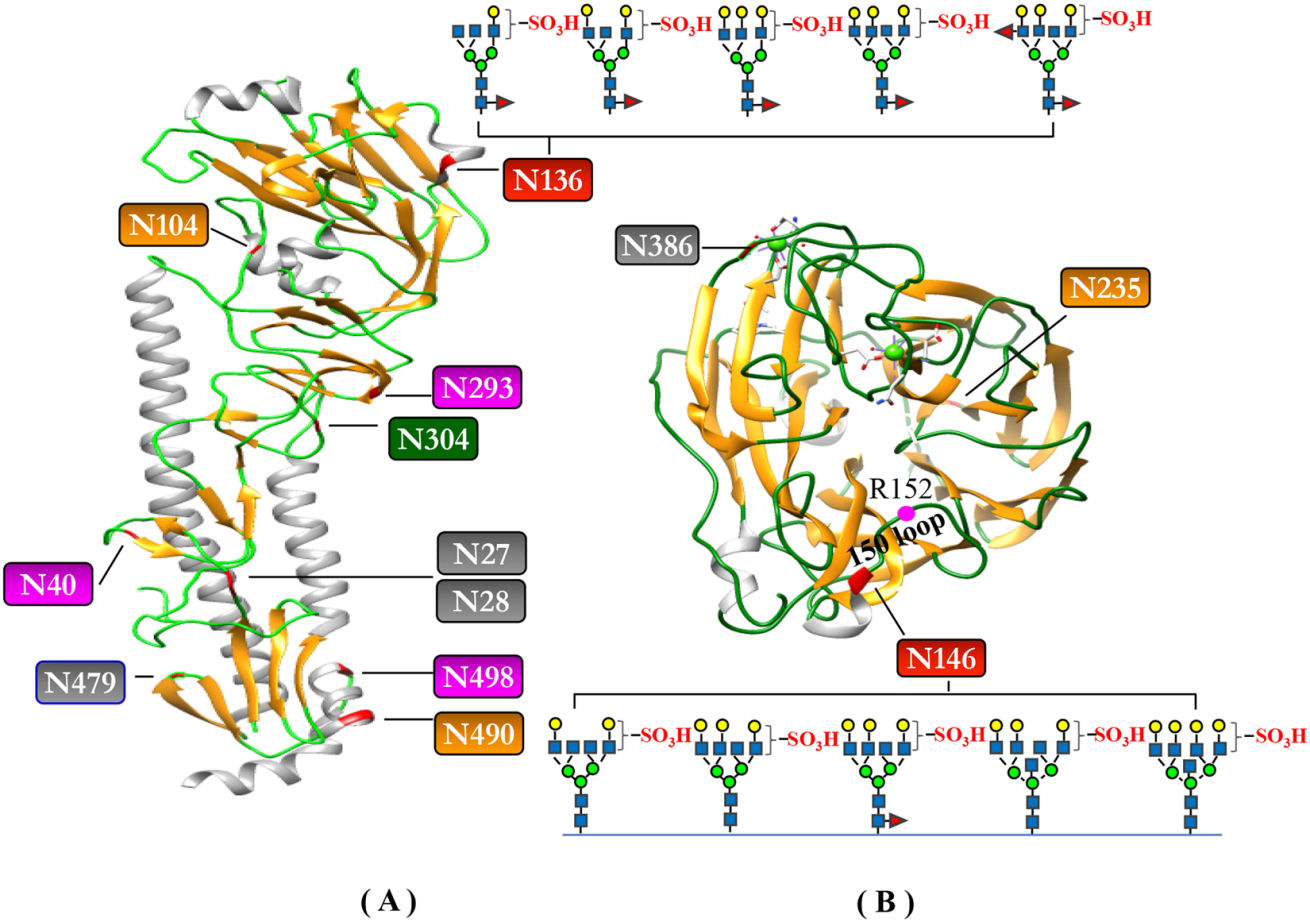
Topological location of N-glycosylation sites of the influenza proteins from the candidate vaccine of NIBRG-121xp. (**A**) hemagglutinin (A/California/04/2009 strain, PDB: 3LZG); (**B**) neuraminidase-receptor (sialic acid) binding region (PDB: 3NSS). The 3D protein structures of influenza HA and NA were visualized using UCSF Chimera program downloaded from the website (https://www.cgl.ucsf.edu). The highly heterogeneous sulfated residues are highlighted in red, whereas the singly glycosylated or non-glycosylated residues with Asn-X-Ser/Thr motif are labeled in grey. Fucosylated glycans, mannose-type glycans, “normal” hybrid and complex glycans of the residues are coloured in pink, green and orange, respectively.

The crystal structure of HA revealed a cylindrical homotrimer with two distinct domains including a globular head of antiparallel β-sheets and a stem loop of triple-stranded α-helices (Fig. 5A)^20^. The mutated site at Asn136 of the influenza vaccine NIBRG-121xp was localized at the head region containing a sialic acid receptor binding cavity which is surrounded by five antigenic sites as described previously^22, 46^, and the N-glycans at Asn136 nearby thus potentially have a great impact on receptor binding to host cells and neutralizing antibodies to antigenic epitopes. Conversely, the presence of a single complex-type glycan or non-glycosylation at sites Asn27, Asn28 and Asn479 is indicative of two embedding regions in the stem loop that might be buried in the structure of HA protein trimer. The absence of glycosylation at Asn557 is consistent with the prediction of its location in a transmembrane domain at the C-terminus and the lipid (palmitate and stearate) embedding region of cysteine residues^28–30^. Surprisingly, two distinct types of *N*-glycans were observed at the neighboring residues Asn490 and Asn498, both positioned on the closely related HA α-helical stem region near the C-terminus with hybrid-type and fucosylated complex-type N-glycans, respectively. Compared to the hybrid and complex glycan chains at residues Asn27, Asn28, Asn40, Asn104, Asn136, Asn293, Asn490 and Asn498, only high-mannose type of glycans were found at Asn304. Asn304 is located on the stem loop region of the internal protein structure. These glycans may be hindered in the structural contact region of the protein subunits and the interaction with the processing enzymes and glycosyltransferases necessary to form hybrid and complex N-glycans.

The structure of each NA is composed of a globular head domain forming a homotetramer containing six four-stranded β-sheets and three α-helices in each monomer (Fig. 5B)^23, 25^. There are three critical glycosylation sites localized at the 150 loop (residues 147 to 152) and 330 loop regions (residues 292–371) of the sialic acid binding cavity. The negatively charged N-glycan sulfation at Asn146 surrounding the 150 loop region is in close proximity to the enzyme active site and a positively charged arginine residue (Arg152). Since the glycosylation of Asn235 is positioned at the opposite side and below the 330 loop region, the identified glycopeptide structures which contain both high-mannose Man_4–8_GlcNAc_2_ and complex-type N-glycans, are expected to have little effect on the enzyme activity. The residue Asn386 at the glycosylation motif of Asn-Phe-Ser is occupied by a single biantennary complex-type glycan of Gal_2_GlcNAc_2_Man_3_GlcNAc_2_, as the extension of the N-glycan is inhibited by its location in the calcium-binding region.

## Discussion

The biosynthesis of N-glycans is initiated by the transfer of a dolichol lipid-linked oligosaccharide precursor (Glc_3_Man_9_GlcNAc_2_) to asparagine residues of the structural motif Asn-X-Ser/Thr/Cys of nascent proteins in the endoplasmic reticulum (ER) by oligosaccharyltransferase^4^. The terminal glucoses and mannoses are then sequentially trimmed by the ER glucosidases and mannosidases to generate high-mannose-type N-glycans (Man_5–9_GlcNAc_2_). Through ER quality control, misfolded proteins are targeted and translocated for ER associated degradation (ERAD) while correctly folded glycoproteins are transported to the Golgi, plasma membrane and other subcellar compartments followed by addition of N-acetylglucosamine and fucose by N-acetylglucosaminyltransferase (GNT1) and fucosyltransferase (α1,3 FucT) to form hybrid, complex and paucimannose-type N-glycans in mature proteins. This results in different types of N-glycans which are often organism, tissue and cell specific. The presence of distinct N-glycans on different sites relies on the protein surface accessibility and the intermolecular interaction with the processing enzymes and glycosyltransferases.

N-glycosylation of HA varies amongst different strains and subtypes due to the lack of conserved glycosylation sites. Addition or deletion of a functional glycosylation site has been shown to have a dramatic effect on protein stability, substrate specificity, virus growth rate, and vaccine yield^2, 3, 47^. Our full-length protein sequence analyses of HA by high-resolution mass spectrometry identified 9 putative N-glycosylation sites in the influenza vaccine NIBRG-121xp, which were modified by high-mannose, hybrid and complex glycans at residues Asn27, Asn28, Asn40, Asn104, Asn136, Asn293, Asn304, Asn490, Asn498. No glycopeptide was detected at residue Asn4679 or Asn557 even after exhaustive analysis using three enzymatic digestion approaches, suggesting that these two sites are either not occupied or occupied at low frequency. Similar MS/MS measurements on the NA determined the N-glycan modifications of 6 residues at Asn50, Asn58, Asn68, Asn146, Asn235 and Asn386 (Supplementary Information Tables S1–S5) that are involved in loop regions of the protein structures (Fig. 5B)^23, 26^. The detection of all glycopeptides showed no sialylated N-glycan branch of influenza proteins in the two vaccines examined, probably due to the desialylation function of the viral NA. The phenomena is in agreement with the previous reports on a number of influenza viruses and vaccines including influenza A/PR/8/34(H1N1)^9^, influenza B/Malaysia/2506/2004^14^, A/Wisconsin/67/2005(H3N2)^10^, rgA/Vietnam/1203/2004(H5N1), A/Indonesia/05/2005 and A/Bar-headed goose/Qinghai Lake/1A/05(H5N1)^48^ derived from mammalian cell culture or those grown in embryonated chicken eggs.

Sulfation at antennal N-acetylglucosamine residues is a rare modification of glycoproteins whose role in the protein structure and the enzyme activity will require further study. The specific sulfoglycosylation site of Asn146 is highly conserved among neuraminidases, and differs from the other glycosylation sites in the NA^39^. Likewise, the N-glycan sulfation was also found specifically in the multi-branched complex-type glycans at Asn136 of HA; these were also fucosylated. The sulfated N-glycans were also predicted and observed previously in the influenza HA proteins from the influenza B/Malaysia/2506/2004^14^, A/Tokyo/3/67^39^, A/Vietnam/1203/2004 (H5N1)^15^, rgA/Vietnam/1203/2004 (H5N1), A/Indonesia/05/2005 (H5N1), A/Bar-headed goose/Qinghai Lake/1A/05(H5N1)^48^. It is worth noting that the sulfation on the designed GlcNAc-type glycan structure may differ from that of influenza A/Tokyo/3/67 at Asn146 containing GalNAc-type glycans^39^. The regulatory role of sulfated N-glycan portion was illustrated by the dramatic decrease of NA activity of the influenza A/WSN/33 virus at the Glyc + mutant versus its wild-type (wt WSN) in the absence of Asn146, *i.e*. loss of the sulfated N-glycans^40^, which is consistent with that of the high-yield candidate vaccine viruses (NIBRG-121xp and NYMC-X181 A) (data not shown). Another study on influenza virus A/Hokkaido/11/2002 (H1N1) in the overexpression of sulfotransferase of Madin Darby Canine Kidney (MDCK) cells to form sulfated GlcNAc structures revealed sulfated glycans increased viral production by 70-fold (48h post-infection) and 13-fold (72h post-infection) compared with the control samples^41^. Due to the lack of a convenient analytical method to selectively identify the structures of sulfated glycans found in low incidence, the determination of substrate specificity of the sufotransferase would be rather difficult in a large proteomics scale^49^. Through LC MS/MS analyses of the two high-yield influenza vaccines, we have reliably identified the N-glycan sulfation sites occurring at highly branched structures which localize on the tri- and tetra-antennary complex-type glycans, but not high-mannose, hybrid and bi-antennary complex glycans. The selective targeting by sulfotransferases and unique location of such sulfated glycans at each active site of two highly expressed influenza proteins suggests a determinant role to regulate protein expression and the enzyme activity.

The additional attachments of tetra-antennary hybrid-type N-glycans and a bisecting GlcNAc to the sulfated complex N-glycans were identified at position Asn136 of HA and Asn146 of NA, respectively. The unusual tetra-antennary hybrid-type N-glycans were only observed in chicken egg glycans and the specific function is unknown. Bisecting GlcNAc is a common termination signal so that the branch is no longer extended, whereas otherwise GlcNAc branches are usually elongated by Gal and Sial. Bisected GlcNAc N-glycans have a wide range of functions as reported previously^50–52^, although the molecular mechanism underlying the protein trafficking and cell adhesion of HA and NA that are regulated by bisecting GlcNAc is still far from clear. Our data are consistent with the notion that bisected N-glycans are localized at the active site of NA surrounding the sialic acid binding cavities. The finding of the modifications, in the region, in high-yielding vaccines may thus suggest a potential role for the bisecting N-glycan modulation as a control of N-glycan branching in the dynamic structure of the targeted proteins.

In conclusion, we have provided a detailed structural study of N-glycans of the influenza proteins in two monovalent influenza vaccines derived from the highly expressed virus strains of NIBRG-121xp and NYMC-X181 A, and identified the novel N-glycan sulfation, fucosylation and bisecting GlcNAc at unique locations in these two proteins (HA and NA). The topological location of N-glycans is highly correlated to the spatial protein structure and the residential ligand/receptor binding. To our knowledge, the glycan structures of NA in influenza virus or vaccine have not been previously reported, possibly due to their low expression level. Structural characterization of glycosylation of influenza antigens in vaccine production is important for evaluating the quality and immunogenicity of vaccines. However, the high complexity of N-glycans remains a challenging problem for the separation and structural identification of those glycan isoforms, even though a wide range of strategies and techniques have developed for analyzing intact glycoproteins, glycopeptides and released carbohydrates. A thorough understanding of the structure and function of N-glycans in commercial vaccines is also desired. Our preliminary finding suggests that site-specific sulfation, core and antennae fucosylation and bisecting GlcNAc of N-glycans of cell surface glycoproteins, hemagglutinin and neuraminidase, may serve critical roles in dynamically regulating the protein structure and the biosynthesis of influenza virus. Future investigations to establish a highly efficient method for analysis of these health products, thus, could assist in the rapid evaluation of the safety and quality control of vaccine production, particularly the development of new strategies to improve the growth yield of influenza vaccines.

## Materials and Methods

### Chemical reagents and enzymes

Hydroxylamine, dithiothreitol (DTT), iodoacetamide, ammonium bicarbonate (NH_4_HCO_3_), formic acid (FA), trifluoroacetic acid (TFA), acetonitrile (ACN), chymotrypsin, proteinase K and pepsin were purchased from Sigma-Aldrich (Oakville, ON, USA). Sequence-grade bovine trypsin was obtained from Roche Diagnostics Corporation (Indianapolis, IN, USA). Porous graphitic carbon (PGC) cartridges were obtained from Mandel Scientific Corporation (Guelph, ON).

### Purification of influenza proteins

Egg-derived monovalent influenza vaccines derived (inactivated, split virion) from the virus strains of NIBRG-121xp (HA: K136N, D239G; NA: N88G) and NYMC-X181A (HA: K236T, Q240R) were collected as reported previously^3, 5, 6^. The viral proteins were purified by centrifugal filtration with the candidate vaccines, and sequentially treated with 10 mM DTT, hydroxylamine solution (Sigma, 2 M, pH 7.4) to remove conjugated lipids from HA followed by alkylation of cysteines with 55 mM iodoacetamide. The samples were further dialyzed against 5 mM NH_4_HCO_3_ and dried in a Savant vacuum centrifuge (Thermo Fisher Scientific, ON, Canada).

### Enzymatic digestion of viral proteins and LTQ-FT mass spectrometric analysis

Aliquots (∼20 µg) of the purified influenza proteins were reconstituted in 20 µL of 25 mM NH_4_HCO_3_ (pH 7.6) or 10 mM Tris buffer (pH 8.0) and digested using trypsin, endoproteinase Asp-N, chymotrypsin, proteinase K and pepsin at 37 °C for 4h as described previously^6^. To check consistency, protein digestions were repeated and samples prepared on different dates for LC MS/MS analyses. In addition, the glycopeptides from protein digestions by trypsin and proteinase K were selectively purified using PGC cartridges which were sequentially washed with 3 mL of 80% (v/v) ACN followed by 3 mL of water, then loaded with the protein digest, washed and eluted by 25% ACN, 50% ACN, and 75% ACN in 0.1% TFA. The fractions were dried using a SpeedVac (Thermo- Fisher).

Each of the digests was subsequently reconstituted in 0.2% FA and analyzed using a nanoAcquity UPLC (Waters, Milford, MA) coupled with LTQ FT-ICR (Thermo Fisher, San Jose, CA, USA) mass spectrometer using the positive ionization mode. The peptides were trapped with a RP Symmetry C18 column (180 µm id × 20 mm length, 5 µm diameter particles) at 5 µl min^−1^ of solvent A (0.1% FA in water) and separated on a C18 analytical column (100 µm id × 100 mm, 1.7 µm particle diameter, BEH 130) at 400 nl min^−1^, using a linear gradient from 5 to 30% solvent B over 40 min, followed by a linear gradient to 85% of solvent B (0.1% FA in ACN) over 10 min. FT-MS scans were acquired with high resolution (100,000) from m/z 300 to 2000, and low-resolution MS/MS measurements in LTQ mode were obtained by data dependent scans of the top eight most intense precursor ions at multiply charged states of 2+, 3+, and 4+ using 30% normalized collision energy. The automated gain control (AGC) targets were set to 1 × 10^6^ for a signal MS full scan, and 2 × 10^5^ for MS^n^ scan. Multistage MS^3^ spectra were acquired at the high intensity monoglycosylated peptide precursor ions selected from the MS/MS fragments. Dynamic exclusion was enabled for a period of 180 s.

To obtain a highly accurate MS/MS measurement on the neutral loss fragments, the tryptic peptides were also analyzed by Thermo Scientific Orbitrap Fusion coupled with an Easy-nLC 1000 system. The peptides were trapped on an Acclaim PepMap 100 (75 µm id × 2 cm length, 3 µm particle diameter) C_18_ column followed by separation with an Easy-spray PepMap^TM^ RSLC C_18_ column (75 µm id × 15 cm length, 3 µm particle diameter) at the same LC gradient conditions as above. The Orbitrap resolving power was used for the MS survey scan at 120,000 and subsequent CID fragmentations at 60,000, while the AGC target value was set up for MS at 4.0e^5^ and MS/MS at 5.0e^4^, respectively. Data acquisition was performed using either a targeted tMS^2^ mode or the data dependent top-N MS/MS scans with a targeted glycopeptide inclusion. Dynamic exclusion was enabled for a period of 30 s

### Database search and peptide identification

Peptide identification was performed using MASCOT Server (version 2.3.0, Matrix Science, London, UK), and LC MS/MS raw data were searched against the NCBI nonredundant database and an in-house database of human influenza vaccine proteins. The search parameters for mass spectrometric data from samples digested with trypsin were restricted to tryptic peptides with a maximum of two missed cleavages. Data from chymotrypsin and proteinase K digestions were searched allowing for nonspecific enzyme cleavage. Cysteine carbamidomethylation (+57.02146 Da) was designated as a fixed modification, the deamidation of asparagine and glutamine (+0.98402 Da) and methionine oxidation (+15.99492 Da) were considered as variable modifications. Mass tolerances were set up to 10 ppm for the FT-MS ions and 1Da for ion-trap MS/MS fragment ions. Peptide assignments were filtered by an ion score cut off of 20, and the significance threshold was adjusted to 0.001 to achieve a false discovery rate (FDR) of less than 1%. The tandem mass spectra of glycopeptides with the diagnostic ions at m/z 204.08, 366.14 and 528.19 were manually interpreted for identifying glycan structures by CID and peptide sequences through MS^3^ fragmentations, as well as the accurate masses at ~1 ppm.

The mass spectrometry proteomics data have been deposited to the ProteomeXchange Consortium via the PRIDE partner repository with the dataset identifier PXD007037^53^.

## Electronic supplementary material

Supplementary Information
