# Supplementary material for: Topological N-glycosylation and site-specific N-glycan sulfation of influenza proteins in the highly expressed H1N1 candidate vaccines
Source: Sci Rep. 2017 Aug 31;7:10232. doi: 10.1038/s41598-017-10714-2 (PMC5579265; doi:10.1038/s41598-017-10714-2)
Supplement: Supplementary file 1 — Supplementary Information [file 41598_2017_10714_MOESM1_ESM.pdf]

Supplementary Information for

Topological N-glycosylation and site-specific sulfation of influenza proteins in the highly expressed H1N1 candidate vaccines

Yi-Min She, Aaron Farnsworth, Xuguang Li and Terry D. Cyr*

Centre for Biologics Evaluation, Biologics and Genetic Therapies Directorate, Health Canada, Ottawa, Ontario K1A 0K9, Canada

```
NIBRG-121xp_HA   MKAILVVLLYTFATANADTLCIGYHANNSTDTVDTVLEKNVTVTHSVNLL 50
                 ||||||||||||||||||||||||||||||||||||||||||||||||||
NYMC-X181A_HA    MKAILVVLLYTFATANADTLCIGYHANNSTDTVDTVLEKNVTVTHSVNLL 50

NIBRG-121xp_HA   EDKHNGKLCKLRGVAPLHLGKCNIAGWILGNPECESLSTASSWSYIVETS 100
                 ||||||||||||||||||||||||||||||||||||||||||||||||||
NYMC-X181A_HA    EDKHNGKLCKLRGVAPLHLGKCNIAGWILGNPECESLSTASSWSYIVETS 100

NIBRG-121xp_HA   SSDNGTCYPGDFIDYEELREQLSSVSSFERFEIFPNTSSWPNHDSNKGVT 150
                 |||||||||||||||||||||||||||||||||||.|||||||||:||||
NYMC-X181A_HA    SSDNGTCYPGDFIDYEELREQLSSVSSFERFEIFPKTSSWPNHDSDKGVT 150

NIBRG-121xp_HA   AACPHAGAKSFYKNLIWLVKKGNSYPKLSKSYINDKGKEVLVLWGIHHPS 200
                 ||||||||||||||||||||||||||||||||||||||||||||||||||
NYMC-X181A_HA    AACPHAGAKSFYKNLIWLVKKGNSYPKLSKSYINDKGKEVLVLWGIHHPS 200

NIBRG-121xp_HA   TSADQQSLYQNADAYVFVGSSRYSKKFKPEIAIRPKVRGQEGRMNYYWTL 250
                 |||||||||||||||||||||||||.||||||||||||.:||||||||||
NYMC-X181A_HA    TSADQQSLYQNADAYVFVGSSRYSKTFKPEIAIRPKVRDREGRMNYYWTL 250

NIBRG-121xp_HA   VEPGDKITFEATGNLVVPRYAFAMERNAGSGIIISDTPVHDCNTTCQTPK 300
                 ||||||||||||||||||||||||||||||||||||||||||||||||||
NYMC-X181A_HA    VEPGDKITFEATGNLVVPRYAFAMERNAGSGIIISDTPVHDCNTTCQTPK 300

NIBRG-121xp_HA   GAINTSLPFQNIHPITIGKCPKYVKSTKLRLATGLRNVPSIQSRGLFGAI 350
                 ||||||||||||||||||||||||||||||||||||||||||||||||||
NYMC-X181A_HA    GAINTSLPFQNIHPITIGKCPKYVKSTKLRLATGLRNVPSIQSRGLFGAI 350

NIBRG-121xp_HA   AGFIEGGWTGMVDGWYGYHHQNEQGSGYAADLKSTQNAIDEITNKVNSVI 400
                 ||||||||||||||||||||||||||||||||||||||||||||||||||
NYMC-X181A_HA    AGFIEGGWTGMVDGWYGYHHQNEQGSGYAADLKSTQNAIDEITNKVNSVI 400

NIBRG-121xp_HA   EKMNTQFTAVGKEFNHLEKRIENLNKKVDDGFLDIWTYNAELLVLLENER 450
                 ||||||||||||||||||||||||||||||||||||||||||||||||||
NYMC-X181A_HA    EKMNTQFTAVGKEFNHLEKRIENLNKKVDDGFLDIWTYNAELLVLLENER 450

NIBRG-121xp_HA   TLDYHDSNVKNLYEKVRSQLKNNAKEIGNGCFEFYHKCDNTCMESVKNGT 500
                 ||||||||||||||||||||||||||||||||||||||||||||||||||
NYMC-X181A_HA    TLDYHDSNVKNLYEKVRSQLKNNAKEIGNGCFEFYHKCDNTCMESVKNGT 500

NIBRG-121xp_HA   YDYPKYSEEAKLNREEIDGVKLESTRIYQILAIYSTVASSLVLVVSLGAI 550
                 ||||||||||||||||||||||||||||||||||||||||||||||||||
NYMC-X181A_HA    YDYPKYSEEAKLNREEIDGVKLESTRIYQILAIYSTVASSLVLVVSLGAI 550

NIBRG-121xp_HA   SFWMCSNGSLQCRICI 566
                 ||||||||||||||||
NYMC-X181A_HA    SFWMCSNGSLQCRICI 566
```

Fig. S1 Local sequence alignment of hemagglutinin (HA) derived from the candidate vaccines of NIBRG-121xp and NYMC-X181A. EMBOSS Water analysis of two protein sequences (http://www.ebi.ac.uk/Tools/psa/emboss_water) of HA showed 99.12% of amino acid identities.

```
NIBRG-121xp_NA   MNPNQKIITIGSVCMTIGMANLILQIGNIISIWISHSIQLGNQNQIETCN 50
                 ||||||||||||||||||||||||||||||||||||||||||||||||||
NYMC-X181A_NA    MNPNQKIITIGSVCMTIGMANLILQIGNIISIWISHSIQLGNQNQIETCN 50

NIBRG-121xp_NA   QSVITYENNTWVNQTYVNISNTNFAAGQSVVSVKLAGGSSLCPVSGWAIY 100
                 |||||||||||||||||||||||||||||||||||||.||||||||||||
NYMC-X181A_NA    QSVITYENNTWVNQTYVNISNTNFAAGQSVVSVKLAGNSSLCPVSGWAIY 100

NIBRG-121xp_NA   SKDNSVRIGSKGDVFVIREPFISCSPLECRTFFLTQGALLNDKHSNGTIK 150
                 ||||||||||||||||||||||||||||||||||||||||||||||||||
NYMC-X181A_NA    SKDNSVRIGSKGDVFVIREPFISCSPLECRTFFLTQGALLNDKHSNGTIK 150

NIBRG-121xp_NA   DRSPYRTLMSCPIGEVPSPYNSRFESVAWSASACHDGINWLTIGISGPDN 200
                 ||||||||||||||||||||||||||||||||||||||||||||||||||
NYMC-X181A_NA    DRSPYRTLMSCPIGEVPSPYNSRFESVAWSASACHDGINWLTIGISGPDN 200

NIBRG-121xp_NA   GAVAVLKYNGIITDTIKSWRNNILRTQESECACVNGSCFTVMTDGPSNGQ 250
                 ||||||||||||||||||||||||||||||||||||||||||||||||||
NYMC-X181A_NA    GAVAVLKYNGIITDTIKSWRNNILRTQESECACVNGSCFTVMTDGPSNGQ 250

NIBRG-121xp_NA   ASYKIFRIEKGKIVKSVEMNAPNYHYEECSCYPDSSEITCVCRDNWHGSN 300
                 ||||||||||||||||||||||||||||||||||||||||||||||||||
NYMC-X181A_NA    ASYKIFRIEKGKIVKSVEMNAPNYHYEECSCYPDSSEITCVCRDNWHGSN 300

NIBRG-121xp_NA   RPWVSFNQNLEYQIGYICSGIFGDNPRPNDKTGSCGPVSSNGANGVKGFS 350
                 ||||||||||||||||||||||||||||||||||||||||||||||||||
NYMC-X181A_NA    RPWVSFNQNLEYQIGYICSGIFGDNPRPNDKTGSCGPVSSNGANGVKGFS 350

NIBRG-121xp_NA   FKYGNGVWIGRTKSISSRNGFEMIWDPNGWTGTDNNFSIKQDIVGINEWS 400
                 ||||||||||||||||||||||||||||||||||||||||||||||||||
NYMC-X181A_NA    FKYGNGVWIGRTKSISSRNGFEMIWDPNGWTGTDNNFSIKQDIVGINEWS 400

NIBRG-121xp_NA   GYSGSFVQHPELTGLDCIRPCFWVELIRGRPKENTIWTSGSSISFCGVNS 450
                 ||||||||||||||||||||||||||||||||||||||||||||||||||
NYMC-X181A_NA    GYSGSFVQHPELTGLDCIRPCFWVELIRGRPKENTIWTSGSSISFCGVNS 450

NIBRG-121xp_NA   DTVGWSWPDGAELPFTIDK 469
                 |||||||||||||||||||
NYMC-X181A_NA    DTVGWSWPDGAELPFTIDK 469
```

Fig. S2 Local sequence alignment of neuraminidase (NA) derived from the candidate vaccines of NIBRG-121xp and NYMC-X181A. EMBOSS Water analysis of two protein sequences (http://www.ebi.ac.uk/Tools/psa/emboss_water) of NA showed 99.79% of amino acid identities.

NIBRG-121xp, HA (566 aa, 11 NxS/T)

```
001 MKAILVVLLY TFATANADTL CIGYHANNST DTVDTVLEKN VTVTHSVNLL EDKHNGKLCK
061 LRGVAPLHLG KCNIAGWILG NPECESLSTA SSWSYIVETS SSDNGTCYPG DFIDYEELRE
121 QLSSVSSFER FEIFPNTSSW PNHDSNKGVT AACPHAGAKS FYKNLIWLVK KGNSYPKLSK
181 SYINDKGKEV LVLWGIHHPS TSADQQSLYQ NADAYVFVGS SRYSKKFKPE IAIRPKVRGQ
241 EGRMNYYWTL VEPGDKITFE ATGNLVVPRY AFAMERNAGS GIIISDTPVH DCNTTCQTPK
301 GAINTSLPFQ NIHPITIGKC PKYVKSTKLR LATGLRNVPS IQSRGLFGAI AGFIEGGWTG
361 MVDGWYGYHH QNEQGSGYAA DLKSTQNAID EITNKVNSVI EKMNTQFTAV GKEFNHLEKR
421 IENLNKKVDD GFLDIWTYNA ELLVLLENER TLDYHDSNVK NLYEKVRSQL KNNAKEIGNG
481 CFEFYHKCDN TCMESVKNGT YDYPKYSEEA KLNREEIDGV KLESTRIYQI LAIYSTVASS
541 LVLVVSLGAI SFWMCSNGSL QCRICI
```

NYMC-X181A, HA (566 aa, 10 NxS/T)

```
001 MKAILVVLLY TFATANADTL CIGYHANNST DTVDTVLEKN VTVTHSVNLL EDKHNGKLCK
061 LRGVAPLHLG KCNIAGWILG NPECESLSTA SSWSYIVETS SSDNGTCYPG DFIDYEELRE
121 QLSSVSSFER FEIFPKTSSW PNHDSDKGVT AACPHAGAKS FYKNLIWLVK KGNSYPKLSK
181 SYINDKGKEV LVLWGIHHPS TSADQQSLYQ NADAYVFVGS SRYSKTFKPE IAIRPKVRDR
241 EGRMNYYWTL VEPGDKITFE ATGNLVVPRY AFAMERNAGS GIIISDTPVH DCNTTCQTPK
301 GAINTSLPFQ NIHPITIGKC PKYVKSTKLR LATGLRNVPS IQSRGLFGAI AGFIEGGWTG
361 MVDGWYGYHH QNEQGSGYAA DLKSTQNAID EITNKVNSVI EKMNTQFTAV GKEFNHLEKR
421 IENLNKKVDD GFLDIWTYNA ELLVLLENER TLDYHDSNVK NLYEKVRSQL KNNAKEIGNG
481 CFEFYHKCDN TCMESVKNGT YDYPKYSEEA KLNREEIDGV KLESTRIYQI LAIYSTVASS
541 LVLVVSLGAI SFWMCSNGSL QCRICI
```

NIBRG-121xp, NA (469 aa; 7 NxS/T)

```
001 MNPNQKIITI GSVCMTIGMA NLILQIGNII SIWISHSIQL GNQNQIETCN QSVITYENNT
061 WVNQTYVNIS NTNFAAGQSV VSVKLAGGSS LCPVSGWAIY SKDNSVRIGS KGDVFVIREP
121 FISCSPLECR TFFLTQGALL NDKHSNGTIK DRSPYRTLMS CPIGEVPSPY NSRFESVAWS
181 ASACHDGINW LTIGISGPDN GAVAVLKYNG IITDTIKSWR NNILRTQESE CACVNGSCFT
241 VMTDGPSNGQ ASYKIFRIEK GKIVKSVEMN APNYHYEECS CYPDSSEITC VCRDNWHGSN
301 RPWVSFNQNL EYQIGYICSG IFGDNPRPND KTGSCGPVSS NGANGVKGFS FKYGNGVWIG
361 RTKSISSRNG FEMIWDPNGW TGTDNNFSIK QDIVGINEWS GYSGSFVQHP ELTGLDCIRP
421 CFWVELIRGR PKENTIWTSG SSISFCGVNS DTVGWSWPDG AELPFTIDK
```

NYMC-X181A, NA (469 aa; 8 NxS/T)

```
001 MNPNQKIITI GSVCMTIGMA NLILQIGNII SIWISHSIQL GNQNQIETCN QSVITYENNT
061 WVNQTYVNIS NTNFAAGQSV VSVKLAGNSS LCPVSGWAIY SKDNSVRIGS KGDVFVIREP
121 FISCSPLECR TFFLTQGALL NDKHSNGTIK DRSPYRTLMS CPIGEVPSPY NSRFESVAWS
181 ASACHDGINW LTIGISGPDN GAVAVLKYNG IITDTIKSWR NNILRTQESE CACVNGSCFT
241 VMTDGPSNGQ ASYKIFRIEK GKIVKSVEMN APNYHYEECS CYPDSSEITC VCRDNWHGSN
301 RPWVSFNQNL EYQIGYICSG IFGDNPRPND KTGSCGPVSS NGANGVKGFS FKYGNGVWIG
361 RTKSISSRNG FEMIWDPNGW TGTDNNFSIK QDIVGINEWS GYSGSFVQHP ELTGLDCIRP
421 CFWVELIRGR PKENTIWTSG SSISFCGVNS DTVGWSWPDG AELPFTIDK
```

Fig. S3 N-Glycosylation motif analysis of the hemagglutinin (HA) and neuraminidase (NA) derived from the candidate vaccines of NIBRG-121xp and NYMC-X181A.

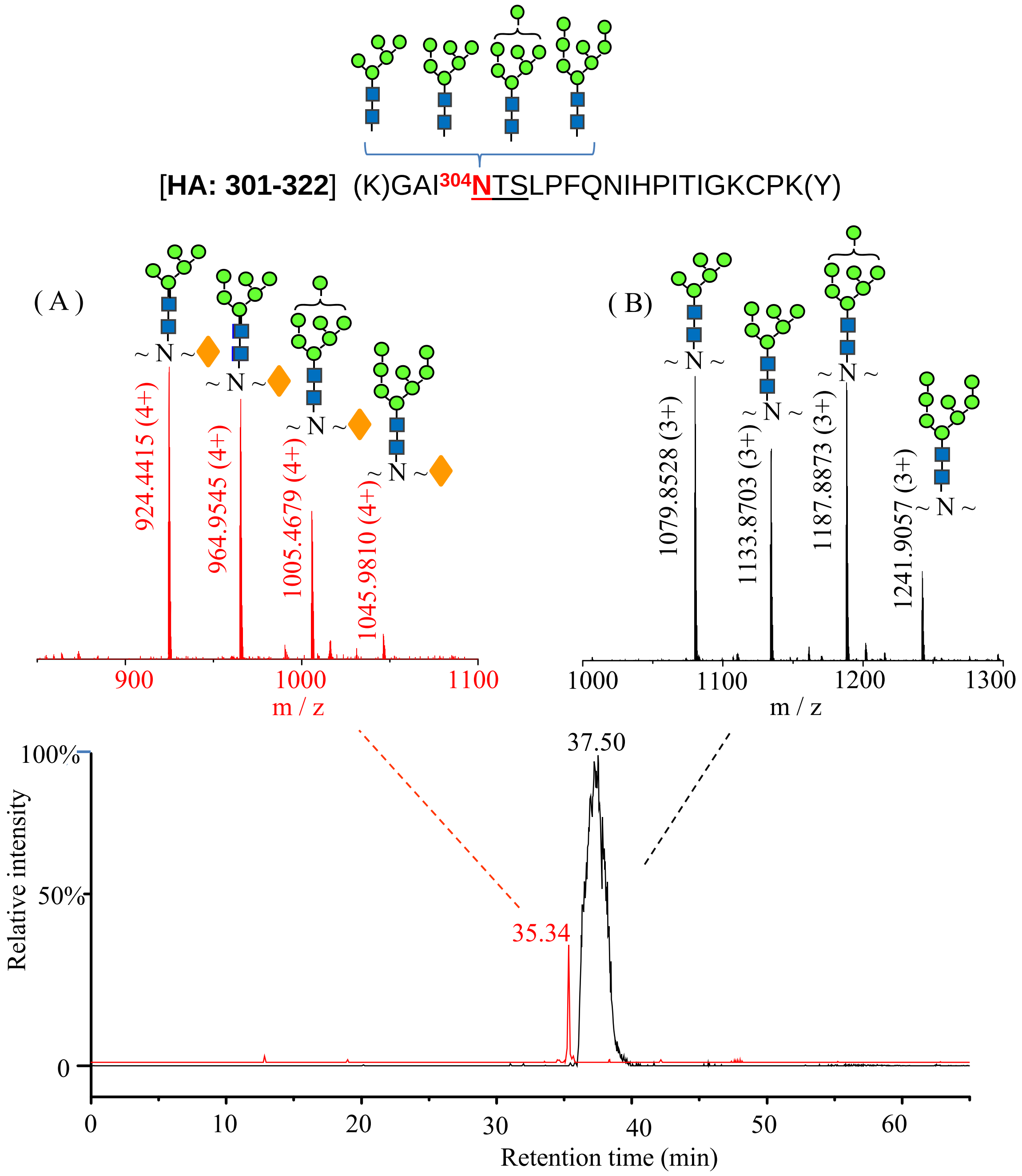

Fig. S4 Sequencing of the tryptic glycopeptide 301-322 with beta-propiolactone modification at Lys319 in the influenza vaccine NIBRG-121xp by LTQ-FT MS/MS. The lower panel illustrates the extracted ion chromatograph of the glycopeptides (coloured in black) and those with additional beta-propiolactone modification (coloured in red). The mass spectrometric profiles are shown in the left inset (A) of β-propiolactone modified glycopeptides with the increase of 72.0211 Da (coloured in red) and the right inset (B) of original glycopeptides (coloured in black)

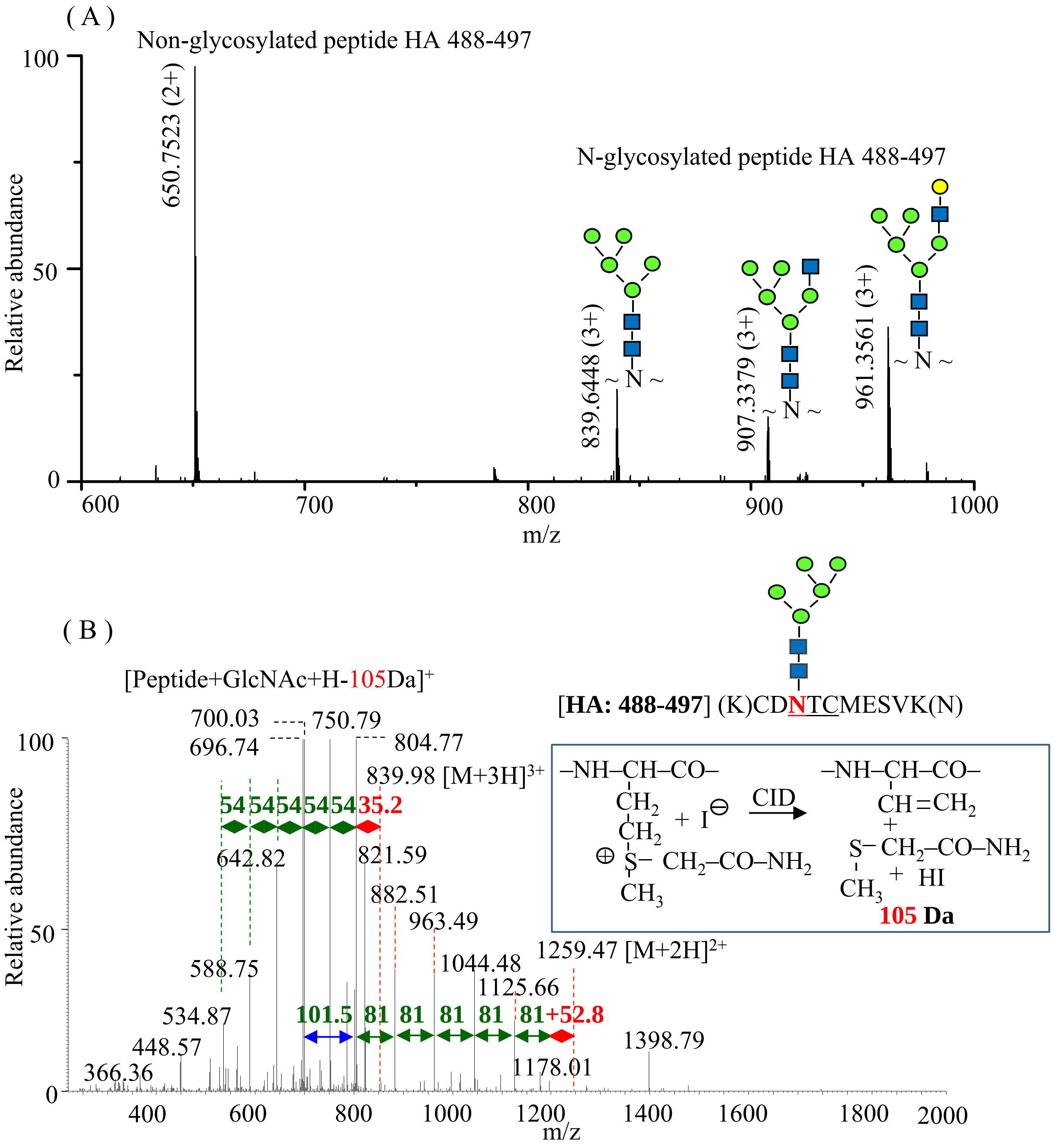

Fig. S5 The N-glycan distribution of the tryptic hemagglutinin peptide 488-497 isolated from the NIBRG-121xp vaccine. (A) LTQ-FT MS spectrum of the peptide with non-glycosylated and N-glycosylated forms. The accurate mass at the doubly charged ion at m/z 650.7523 shows the Cys488-, Cys492- and Met493-carbamidomethylated peptide without glycosylation. (B) Ion-trap MS/MS spectrum of the triply charged ion at m/z 839.98. MS/MS spectrum of the tryptic glycopeptide ion of m/z 839.6448 displays a fragmentation pattern of the Cys488-, Cys492- and Met493-carboxyamidomethylated peptide with high-mannose type N-glycan Man$_5$GlcNAc$_2$, and the fragmentation of iodoacetamide-derivatized methionine yields a neutral loss of 105 Da under low-energy collision dissociation as shown in the insert.

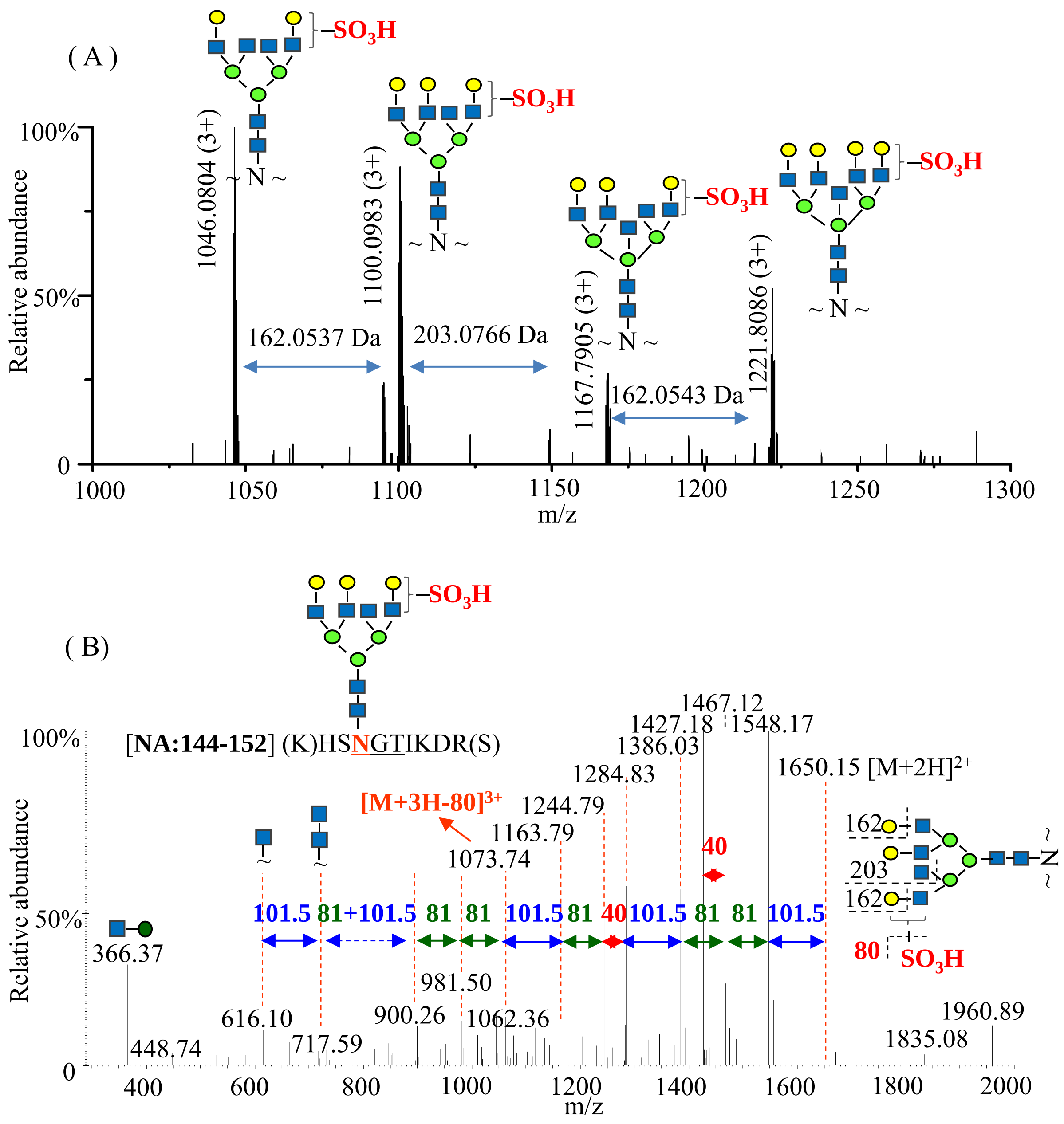

Fig. S6 The N-glycan distribution of tryptic neuraminidase glycopeptides at residues 144-152 isolated from the NIBRG-121xp vaccine. (A) LTQ-FT MS spectrum of the peptides containing sulfated complex-type N-glycans and bisecting GlcNAc forms. (B) Ion-trap MS/MS spectrum of the triply charged ion at m/z 1100.10 (3+). The CID MS/MS spectrum displays a series of consequential loss of neutral saccharides from the glycan structure, and the m/z difference of 40 units between the doubly ion fragments (*i.e.* 80 Da) is caused by the loss of a sulfated group at the branched N-glycan side chain.

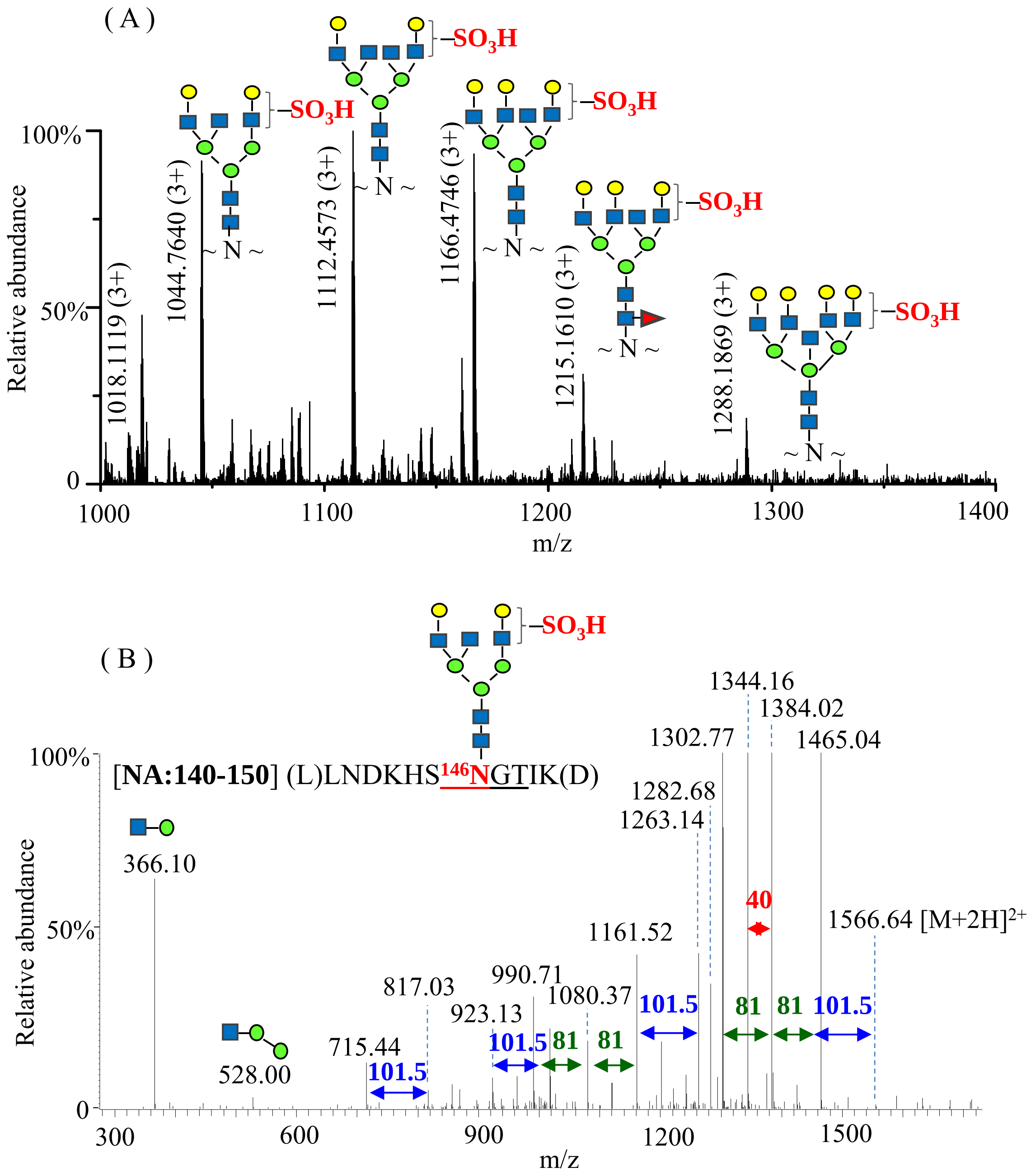

Fig. S7 The N-glycan distribution of the neuraminidase peptide at residues 140-150 isolated from the NIBRG-121xp vaccine, resulting from two enzymatic digestions by trypsin followed by chymotrypsin. (A) LTQ-FT MS spectrum of the peptide with sulfated complex-type N-glycan and bisected GlcNAc forms. (B) Ion-trap MS/MS spectrum of the triply charged ion at m/z 1044.76 (3+). The CID MS/MS spectrum displays a series of sequential loss of neutral saccharides from the glycan structure, and the m/z difference of 40 units between two doubly charged daughter ion fragments (*i.e.* 80 Da) is generated from the loss of a sulfated group at the branched N-glycan side chain.

[**HA: 131-138**] (R)FEIFPI[136]NTS(S)

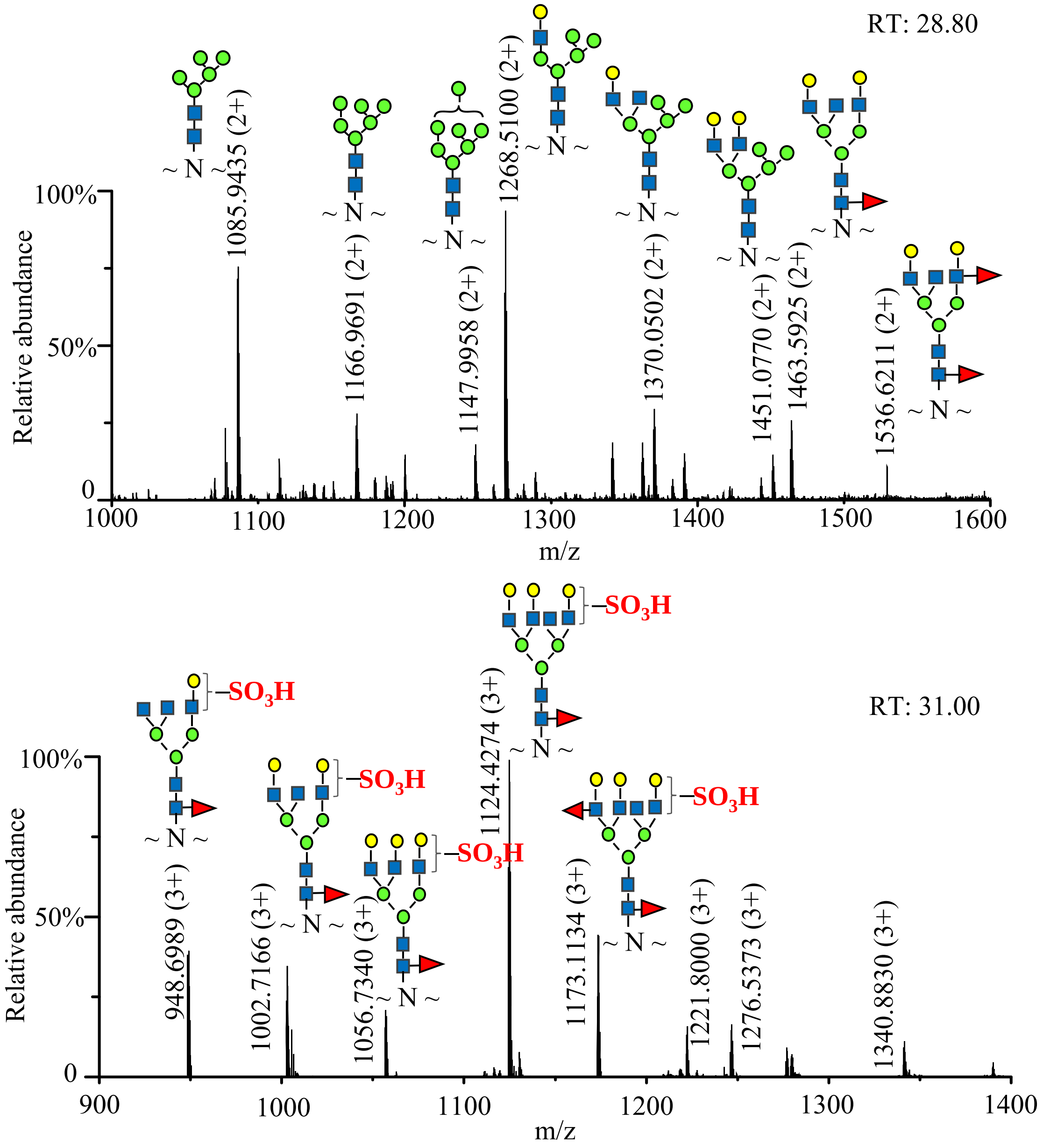

Fig. S8 The N-glycan distribution of sulfated and fucosylated glycopeptides at residues 131-138 of hemagglutinin isolated from the enzymatic digest of the NIBRG-121xp vaccine with trypsin followed by proteinase K.

Table S1. Identification of the glycopeptides of influenza proteins in the vaccine of NIBRG-121xp by tryptic digestion and LTQ-FT MS/MS analyses

Glycopeptide m/z (charge)	Meas. MH+	Calc. MH+	ppm	Peptide MH+	Peptide sequence [protein: residue position]	Glycan mass	Glycan composition	Glycan structure
1079.4530(3+)	3236.3433	3236.3424	0	2019.9195	**[HA: 131-147]** (R)FEIFP^{136}NTSSWPNHDSNK(G)	1216.4229	Man$_5$(GlcNAc)$_2$	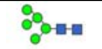
1133.4706(3+)	3398.3961	3398.3952	0	2019.9195	**[HA: 131-147]** (R)FEIFP^{136}NTSSWPNHDSNK(G)	1378.4757	Man$_6$(GlcNAc)$_2$	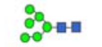
1201.1639(3+)	3601.4760	3601.4746	0	2019.9195	**[HA: 131-147]** (R)FEIFP^{136}NTSSWPNHDSNK(G)	1581.5551	Gal(GlcNAc)Man$_5$(GlcNAc)$_2$	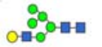
1268.8566(3+)	3804.5541	3804.5539	0	2019.9195	**[HA: 131-147]** (R)FEIFP^{136}NTSSWPNHDSNK(G)	1784.6344	(GlcNAc)$_2$Man$_6$(GlcNAc)$_2$	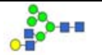
1322.8733(3+)	3966.6042	3966.6068	1	2019.9195	**[HA: 131-147]** (R)FEIFP^{136}NTSSWPNHDSNK(G)	1946.6873	(GlcNAc)$_2$Man$_7$(GlcNAc)$_2$	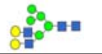
1268.2105(3+)	3802.6159	3802.6152	0	2586.1923	**[HA:277-300]** (R)NAGSGIIISDTPVHDC^{293}NTTCQTPK(G)	1216.4229	Man$_5$(GlcNAc)$_2$	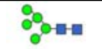
1389.9215(3+)	4167.7489	4167.7474	0	2586.1923	**[HA:277-300]** (R)NAGSGIIISDTPVHDC^{293}NTTCQTPK(G)	1581.5551	Gal(GlcNAc)Man$_5$(GlcNAc)$_2$	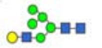
1438.6056(3+)	4313.8012	4313.8053	-1	2586.1923	**[HA:277-300]** (R)NAGSGIIISDTPVHDC^{293}NTTCQTPK(G)	1727.6130	Gal(GlcNAc)Man$_5$Fuc(GlcNAc)$_2$	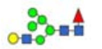
1452.2801(3+)	4354.8247	4354.8318	-2	2586.1923	**[HA:277-300]** (R)NAGSGIIISDTPVHDC^{293}NTTCQTPK(G)	1768.6395	Gal$_2$(GlcNAc)$_2$Man$_3$Fuc(GlcNAc)$_2$	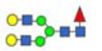
1519.9774(3+)	4557.9166	4557.9112	1	2586.1923	**[HA:277-300]** (R)NAGSGIIISDTPVHDC^{293}NTTCQTPK(G)	1971.7189	Gal$_2$(GlcNAc)$_3$Man$_3$Fuc(GlcNAc)$_2$	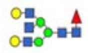
1573.9955(3+)	4719.9703	4719.9640	1	2586.1923	**[HA:277-300]** (R)NAGSGIIISDTPVHDC^{293}NTTCQTPK(G)	2133.7717	Gal$_3$(GlcNAc)$_3$Man$_3$Fuc(GlcNAc)$_2$	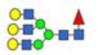
1079.8528(3+)	3237.5427	3237.5407	1	2021.1178	**[HA: 301-319]** (K)GAI^{304}NTSLPFQNIHPITIGK(C)	1216.4229	Man$_5$(GlcNAc)$_2$	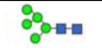
1133.8703(3+)	3399.5952	3399.5935	1	2021.1178	**[HA: 301-319]** (K)GAI^{304}NTSLPFQNIHPITIGK(C)	1378.4757	Man$_6$(GlcNAc)$_2$	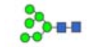
1187.8873(3+)	3561.6462	3561.6463	0	2021.1178	**[HA: 301-319]** (K)GAI^{304}NTSLPFQNIHPITIGK(C)	1540.5285	Man$_7$(GlcNAc)$_2$	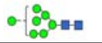
1241.9057(3+)	3723.7014	3723.6992	1	2021.1178	**[HA: 301-319]** (K)GAI^{304}NTSLPFQNIHPITIGK(C)	1702.5814	Man$_8$(GlcNAc)$_2$	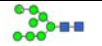
906.4353(4+)	3622.7177	3622.7191	0	2406.2962	**[HA: 301-322]** (K)GAI^{304}NTSLPFQNIHPITIGKCPK(Y)	1216.4229	Man$_5$(GlcNAc)$_2$	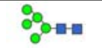
946.9490(4+)	3784.7725	3784.7719	0	2406.2962	**[HA: 301-322]** (K)GAI^{304}NTSLPFQNIHPITIGKCPK(Y)	1378.4757	Man$_6$(GlcNAc)$_2$	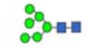
987.4623(4+)	3946.8257	3946.8247	0	2406.2962	**[HA: 301-322]** (K)GAI^{304}NTSLPFQNIHPITIGKCPK(Y)	1540.5285	Man$_7$(GlcNAc)$_2$	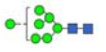
1027.9758(4+)	4108.8797	4108.8776	1	2406.2962	**[HA: 301-322]** (K)GAI^{304}NTSLPFQNIHPITIGKCPK(Y)	1702.5814	Man$_8$(GlcNAc)$_2$	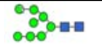
924.4415(4+)	3694.7425	3694.7402	1	2478.3173	**[HA: 301-322]** (K)GAI^{304}NTSLPFQNIHPITIGKCPK(Y) modified by β-propiolactone with 72.0211 Da	1216.4229	Man$_5$(GlcNAc)$_2$	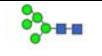
964.9545(4+)	3856.7945	3856.7930	0	2478.3173	**[HA: 301-322]** (K)GAI^{304}NTSLPFQNIHPITIGKCPK(Y) modified by β-propiolactone with 72.0211 Da	1378.4757	Man$_6$(GlcNAc)$_2$	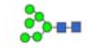
1005.4679(4+)	4018.8481	4018.8458	1	2478.3173	**[HA: 301-322]** (K)GAI^{304}NTSLPFQNIHPITIGKCPK(Y) modified by β-propiolactone with 72.0211 Da	1540.5285	Man$_7$(GlcNAc)$_2$	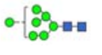
1045.9810(4+)	4180.9005	4180.8987	0	2478.3173	**[HA: 301-322]** (K)GAI^{304}NTSLPFQNIHPITIGKCPK(Y) modified by β-propiolactone with 72.0211 Da	1702.5814	Man$_8$(GlcNAc)$_2$	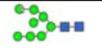
839.6448(3+)	2516.9187	2516.9196	0	1300.4967	**[HA: 488-497]** (K)CD^{490}NTCMESVK(N)	1216.4229	Man$_5$(GlcNAc)$_2$	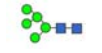
907.3379(3+)	2719.9980	2719.9989	0	1300.4967	**[HA: 488-497]** (K)CD^{490}NTCMESVK(N)	1419.5022	(GlcNAc)Man$_5$(GlcNAc)$_2$	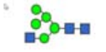
961.3561(3+)	2882.0526	2882.0518	0	1300.4967	**[HA: 488-497]** (K)CD^{490}NTCMESVK(N)	1581.5551	Gal(GlcNAc)Man$_5$(GlcNAc)$_2$	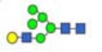
1268.8289(3+)	3804.4710	3804.4702	0	2181.8886	**[HA: 488-505]** (K)CD^{490}NTCMESVK^{498}NGTYDYPK(Y)	1622.5816	Gal$_2$(GlcNAc)$_2$Man$_3$(GlcNAc)$_2$	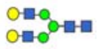
1317.5141(3+)	3950.5266	3950.5281	0	2181.8886	**[HA: 488-505]** (K)CD^{490}NTCMESVK^{498}NGTYDYPK(Y)	1768.6395	Gal$_2$(GlcNAc)$_2$Man$_3$Fuc(GlcNAc)$_2$	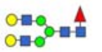
1336.5224(3+)	4007.5515	4007.5496	1	2181.8886	**[HA: 488-505]** (K)CD^{490}NTCMESVK^{498}NGTYDYPK(Y)	1825.6610	Gal$_2$(GlcNAc)$_3$Man$_3$(GlcNAc)$_2$	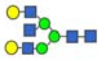

1366.1998(3+)	4096.5837	4096.5860	-1	2181.8886	**[HA: 488-505]** (K)CD^{490}NTCMESVK^{498}NGTYDYPK(Y)	1914.6974	$Gal_2Fuc(GlcNAc)_2Man_3Fuc(GlcNAc)_2$	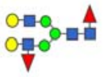
1385.2085(3+)	4153.6098	4153.6075	1	2181.8886	**[HA: 488-505]** (K)CD^{490}NTCMESVK^{498}NGTYDYPK(Y)	1971.7189	$Gal_2(GlcNAc)_3Man_3Fuc(GlcNAc)_2$	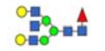
1439.2266(3+)	4315.6641	4315.6603	1	2181.8886	**[HA: 488-505]** (K)CD^{490}NTCMESVK^{498}NGTYDYPK(Y)	2133.7717	$Gal_3(GlcNAc)_3Man_3Fuc(GlcNAc)_2$	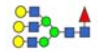
1128.7028(4+)	4511.7877	4511.7828	1	2889.2012	**[HA: 488-511]** (K) CD^{490}N TCMESVK^{498}NGTYDYPK YSEEAK(L)	1622.5816	$Gal_2(GlcNAc)_2Man_3(GlcNAc)_2$	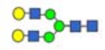
1165.2156(4+)	4657.8389	4657.8407	0	2889.2012	**[HA: 488-511]** (K) CD^{490}N TCMESVK^{498}NGTYDYPK YSEEAK(L)	1768.6395	$Gal_2(GlcNAc)_2Man_3Fuc(GlcNAc)_2$	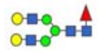
1201.7320(4+)	4803.9045	4803.8986	1	2889.2012	**[HA: 488-511]** (K) CD^{490}N TCMESVK^{498}NGTYDYPK YSEEAK(L)	1914.6974	$Gal_2Fuc(GlcNAc)_2Man_3Fuc(GlcNAc)_2$	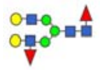
1215.9881(4+)	4860.9289	4860.9201	2	2889.2012	**[HA: 488-511]** (K) CD^{490}N TCMESVK^{498}NGTYDYPK YSEEAK(L)	1971.7189	$Gal_2(GlcNAc)_3Man_3Fuc(GlcNAc)_2$	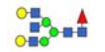
1256.4966(4+)	5022.9629	5022.9729	-2	2889.2012	**[HA: 488-511]** (K) CD^{490}N TCMESVK^{498}NGTYDYPK YSEEAK(L)	2133.7717	$Gal_3(GlcNAc)_3Man_3Fuc(GlcNAc)_2$	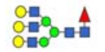
1096.4470(3+)	3287.3253	3287.3255	0	1664.7439	**[HA: 498-511]** (K)^{498}NGTYDYPKYSEEAK(L)	1622.5816	$Gal_2(GlcNAc)_2Man_3(GlcNAc)_2$	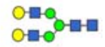
1145.1330(3+)	3433.3833	3433.3834	0	1664.7439	**[HA: 498-511]** (K)^{498}NGTYDYPKYSEEAK(L)	1768.6395	$Gal_2(GlcNAc)_2Man_3Fuc(GlcNAc)_2$	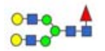
1193.8193(3+)	3579.4422	3579.4413	0	1664.7439	**[HA: 498-511]** (K)^{498}NGTYDYPKYSEEAK(L)	1914.6974	$Gal_2Fuc(GlcNAc)_2Man_3Fuc(GlcNAc)_2$	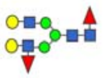
807.3417(3+)	2420.0094	2420.0081	1	756.3999	**[NA:144-150]** (K)HS^{146}NGTIK(D)	1663.6082	$Gal(GlcNAc)_3Man_3(GlcNAc)_2$	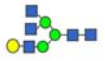
861.3594(3+)	2582.0625	2582.0609	1	756.3999	**[NA:144-150]** (K)HS^{146}NGTIK(D)	1825.6610	$Gal_2(GlcNAc)_3Man_3(GlcNAc)_2$	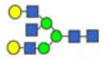
1108.9650(2+)	2216.9222	2216.9287	-3	756.3999	**[NA:144-150]** (K)HS^{146}NGTIK(D)	1460.5288	$Gal(GlcNAc)_2Man_3(GlcNAc)_2$	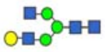
1210.5089(2+)	2420.0100	2420.0081	1	756.3999	**[NA:144-150]** (K)HS^{146}NGTIK(D)	1663.6082	$Gal(GlcNAc)_3Man_3(GlcNAc)_2$	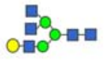
1291.5363(2+)	2582.0648	2582.0609	2	756.3999	**[NA:144-150]** (K)HS^{146}NGTIK(D)	1825.6610	$Gal_2(GlcNAc)_3Man_3(GlcNAc)_2$	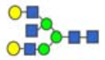
856.0270(3+)	2566.0654	2566.0660	0	756.3999	**[NA:144-150]** (K)HS^{146}NGTIK(D)	1809.6661	$Gal(GlcNAc)_3Man_3Fuc(GlcNAc)_2$	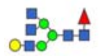
910.0468(3+)	2728.1247	2728.1188	2	756.3999	**[NA:144-150]** (K)HS^{146}NGTIK(D)	1971.7189	$Gal_2(GlcNAc)_3Man_3Fuc(GlcNAc)_2$	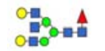
748.6562(3+)	2243.9529	2243.9508	2	1027.5279	**[NA:144-152]** (K)HS^{146}NGTIKDR(S)	1216.4229	$Man_5(GlcNAc)_2$	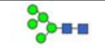
870.3667(3+)	2609.0844	2609.0830	1	1027.5279	**[NA:144-152]** (K)HS^{146}NGTIKDR(S)	1581.5551	$Gal(GlcNAc)Man_5(GlcNAc)_2$	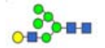
884.0413(3+)	2650.1082	2650.1095	-1	1027.5279	**[NA:144-152]** (K)HS^{146}NGTIKDR(S)	1622.5816	$Gal_2(GlcNAc)_2Man_3(GlcNAc)_2$	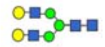
897.7179(3+)	2691.1380	2691.1361	1	1027.5279	**[NA:144-152]** (K)HS^{146}NGTIKDR(S)	1663.6082	$Gal(GlcNAc)_3Man_3(GlcNAc)_2$	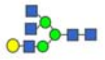
951.7355(3+)	2853.1908	2853.1889	1	1027.5279	**[NA:144-152]** (K)HS^{146}NGTIKDR(S)	1825.6610	$Gal_2(GlcNAc)_3Man_3(GlcNAc)_2$	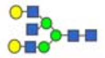
946.4043(3+)	2837.1972	2837.1940	1	1027.5279	**[NA:144-152]** (K)HS^{146}NGTIKDR(S)	1809.6661	$Gal(GlcNAc)_3Man_3Fuc(GlcNAc)_2$	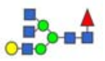
1000.4220(3+)	2999.2503	2999.2468	1	1027.5279	**[NA:144-152]** (K)HS^{146}NGTIKDR(S)	1971.7189	$Gal_2(GlcNAc)_3Man_3Fuc(GlcNAc)_2$	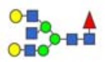
1046.0804(3+)	3136.2256	3136.2251	0	1027.5279	**[NA:144-152]** (K)HS^{146}NGTIKDR(S)	2108.6972	$Gal_2(SO_3)(GlcNAc)_4Man_3(GlcNAc)_2$	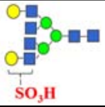

21100.0983(3+)	3298.2793	3298.2779	0	1027.5279	**[NA:144-152]** (K)HS^{146}NGTIKDR(S)	2270.7500	Gal$_3$(SO$_3$)(GlcNAc)$_4$Man$_3$(GlcNAc)$_2$	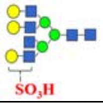
1148.7836(3+)	3444.3352	3444.3358	0	1027.5279	**[NA:144-152]** (K)HS^{146}NGTIKDR(S)	2416.8079	Gal$_3$(SO$_3$)(GlcNAc)$_4$Man$_3$Fuc(GlcNAc)$_2$	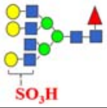
1167.7905 (3+)	3501.3558	3501.3573	0	1027.5279	**[NA:144-152]** (K)HS^{146}NGTIKDR(S)	2473.8294	Gal$_3$(SO$_3$)(GlcNAc)$_5$Man$_3$(GlcNAc)$_2$	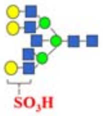
1221.8086 (3+)	3663.4102	3663.4101	0	1027.5279	**[NA:144-152]** (K)HS^{146}NGTIKDR(S)	2635.8822	Gal$_4$(SO$_3$)(GlcNAc)$_5$Man$_3$(GlcNAc)$_2$	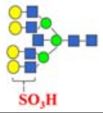

Table S2. Identification of the glycopeptides of influenza proteins in the vaccine of NYMC-X181A by tryptic digestion and LTQ-FT MS/MS analyses

Glycopeptide m/z (charge)	Meas.Mass	Calc. MH+	ppm	Peptide MH+	Peptide sequence [protein: residue position]	Glycan mass	Glycan composition	Glycan Structure
1268.2106(3+)	3802.6162	3802.6152	0	2586.1923	**[HA:277-300]** (R)NAGSGIIISDTPVHDC^{293}NTTCQTPK(G)	1216.4229	(GlcNAc)$_2$Man$_5$	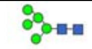
1389.9225(3+)	4167.7519	4167.7474	1	2586.1923	**[HA:277-300]** (R)NAGSGIIISDTPVHDC^{293}NTTCQTPK(G)	1581.5551	Gal(GlcNAc)Man$_5$(GlcNAc)$_2$	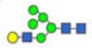
1438.6070(3+)	4313.8054	4313.8053	0	2586.1923	**[HA:277-300]** (R)NAGSGIIISDTPVHDC^{293}NTTCQTPK(G)	1727.6130	Gal(GlcNAc)Man$_5$Fuc(GlcNAc)$_2$	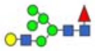
1452.2797(3+)	4354.8235	4354.8318	-2	2586.1923	**[HA:277-300]** (R)NAGSGIIISDTPVHDC^{293}NTTCQTPK(G)	1768.6395	Gal$_2$(GlcNAc)$_2$Man$_3$Fuc(GlcNAc)$_2$	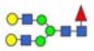
1519.9761(3+)	4557.9127	4557.9112	0	2586.1923	**[HA:277-300]** (R)NAGSGIIISDTPVHDC^{293}NTTCQTPK(G)	1971.7189	Gal$_2$(GlcNAc)$_3$Man$_3$Fuc(GlcNAc)$_2$	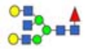
1573.9940(3+)	4719.9664	4719.9640	1	2586.1923	**[HA:277-300]** (R)NAGSGIIISDTPVHDC^{293}NTTCQTPK(G)	2133.7717	Gal$_3$(GlcNAc)$_3$Man$_3$Fuc(GlcNAc)$_2$	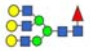
1079.8527(3+)	3237.5425	3237.5407	1	2021.1178	**[HA: 301-319]** (K)GAI^{304}NTSLPFQNIHPITIGK(C)	1216.4229	Man$_5$(GlcNAc)$_2$	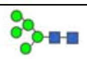
1133.8699(3+)	3399.5941	3399.5935	0	2021.1178	**[HA: 301-319]** (K)GAI^{304}NTSLPFQNIHPITIGK(C)	1378.4757	Man$_6$(GlcNAc)$_2$	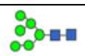
1187.8875(3+)	3561.6468	3561.6463	0	2021.1178	**[HA: 301-319]** (K)GAI^{304}NTSLPFQNIHPITIGK(C)	1540.5285	Man$_7$(GlcNAc)$_2$	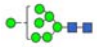
1241.9054(3+)	2723.7005	3723.6992	0	2021.1178	**[HA: 301-319]** (K)GAI^{304}NTSLPFQNIHPITIGK(C)	1702.5814	Man$_8$(GlcNAc)$_2$	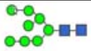
1250.2489(3+)	3748.7311	3748.7308	0	2021.1178	**[HA: 301-319]** (K)GAI^{304}NTSLPFQNIHPITIGK(C)	1727.6130	Gal(GlcNAc)Man$_5$Fuc(GlcNAc)$_2$	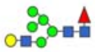
1005.4681(4+)	4018.8489	4018.8458	1	2406.2962	**[HA: 301-322]** (K)GAI^{304}NTSLPFQNIHPITIGKCPK(Y) modified by "72.0211 Da"	1540.5285	Man$_7$(GlcNAc)$_2$	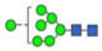
1045.9813(4+)	4180.9017	4180.8987	1	2406.2962	**[HA: 301-322]** (K)GAI^{304}NTSLPFQNIHPITIGKCPK(Y) modified by "72.0211 Da"	1702.5814	Man$_8$(GlcNAc)$_2$	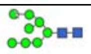
1268.8281(3+)	3804.4687	3804.4702	0	2181.8886	**[HA: 488-505]** (K)CD^{490}NTCMESVK^{498}NGTYDYPK(Y)	1622.5816	Gal$_2$(GlcNAc)$_2$Man$_3$(GlcNAc)$_2$	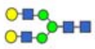
1317.5146(3+)	3950.2815	3950.5281	0	2181.8886	**[HA: 488-505]** (K)CD^{490}NTCMESVK^{498}NGTYDYPK(Y)	1768.6395	Gal$_2$(GlcNAc)$_2$Man$_3$Fuc(GlcNAc)$_2$	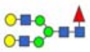
1385.2070(3+)	4153.6054	4153.6075	-1	2181.8886	**[HA: 488-505]** (K)CD^{490}NTCMESVK^{498}NGTYDYPK(Y)	1971.7189	Gal$_2$(GlcNAc)$_3$Man$_3$Fuc(GlcNAc)$_2$	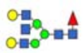
1439.2272(3+)	4315.6660	4315.6603	1	2181.8886	**[HA: 488-505]** (K)CD^{490}NTCMESVK^{498}NGTYDYPK(Y)	2133.7717	Gal$_3$(GlcNAc)$_3$Man$_3$Fuc(GlcNAc)$_2$	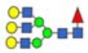
1128.7012(4+)	4511.7813	4511.7828	0	2889.2012	**[HA: 488-511]** (K) CD^{490}N TCMESVK^{498}NGTYDYPK YSEEAK(L)	1622.5816	Gal$_2$(GlcNAc)$_2$Man$_3$(GlcNAc)$_2$	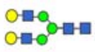
1165.2155(4+)	4657.8385	4657.8407	-1	2889.2012	**[HA: 488-511]** (K) CD^{490}N TCMESVK^{498}NGTYDYPK YSEEAK(L)	1768.6395	Gal$_2$(GlcNAc)$_2$Man$_3$Fuc(GlcNAc)$_2$	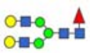

1201.7308(4+)	4803.8997	4803.8986	0	2889.2012	**[HA: 488-511]**<br>(K) CD^{490}N TCMESVK^{498}NGTYDYPK<br>YSEEAK(L)	1914.6974	$Gal_2Fuc(GlcNAc)_2Man_3Fuc(GlcNAc)_2$	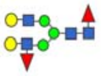
1215.9862(4+)	4860.9213	4860.9201	0	2889.2012	**[HA: 488-511]**<br>(K) CD^{490}N TCMESVK^{498}NGTYDYPK<br>YSEEAK(L)	1971.7189	$Gal_2(GlcNAc)_3Man_3Fuc(GlcNAc)_2$	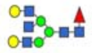
1238.2426(4+)	4949.9469	4949.9565	-2	2889.2012	**[HA: 488-511]**<br>(K) CD^{490}N TCMESVK^{498}NGTYDYPK<br>YSEEAK(L)	2060.7553	$Gal_2Fuc_2(GlcNAc)_2Man_3Fuc(GlcNAc)_2$	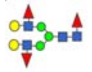
1256.5004(4+)	5022.9781	5022.9729	1	2889.2012	**[HA: 488-511]**<br>(K) CD^{490}N TCMESVK^{498}NGTYDYPK<br>YSEEAK(L)	2133.7717	$Gal_3(GlcNAc)_3Man_3Fuc(GlcNAc)_2$	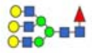
1096.4469(3+)	3287.3251	3287.3255	0	1664.7439	**[HA: 498-511]**<br>(K)^{498}NGTYDYPKYSEEAK(L)	1622.5816	$Gal_2(GlcNAc)_2Man_3(GlcNAc)_2$	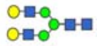
1145.1324(3+)	3433.3816	3433.3834	-1	1664.7439	**[HA: 498-511]**<br>(K)^{498}NGTYDYPKYSEEAK(L)	1768.6395	$Gal_2(GlcNAc)_2Man_3Fuc(GlcNAc)_2$	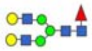
1212.8257(3+)	3636.4615	3636.4628	-3	1664.7439	**[HA: 498-511]**<br>(K)^{498}NGTYDYPKYSEEAK(L)	1971.7189	$Gal_2(GlcNAc)_3Man_3Fuc(GlcNAc)_2$	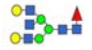
779.9911(3+)	2337.9576	2337.9550	1	756.3999	**[NA:144-150]**<br>(K)HS^{146}NGTIK(S)	1581.5551	$Gal(GlcNAc)Man_5(GlcNAc)_2$	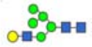
986.9158(2+)	1972.8238	1972.8228	1	756.3999	**[NA:144-150]**<br>(K)HS^{146}NGTIK(S)	1216.4229	$Man_5(GlcNAc)_2$	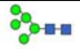
1088.4554(2+)	2175.9030	2175.9021	0	756.3999	**[NA:144-150]**<br>(K)HS^{146}NGTIK(S)	1419.5022	$(GlcNAc)Man_5(GlcNAc)_2$	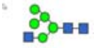
1169.4820(2+)	2337.9562	2337.9550	1	756.3999	**[NA:144-150]**<br>(K)HS^{146}NGTIK(S)	1581.5551	$Gal(GlcNAc)Man_5(GlcNAc)_2$	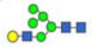
1283.5368(2+)	2566.0658	2566.0660	0	756.3999	**[NA:144-150]**<br>(K)HS^{146}NGTIK(S)	1809.6661	$Gal(GlcNAc)_3Man_3Fuc(GlcNAc)_2$	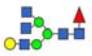
1364.5654(2+)	2728.1230	2728.1188	1	756.3999	**[NA:144-150]**<br>(K)HS^{146}NGTIK(S)	1971.7189	$Gal_2(GlcNAc)_3Man_3Fuc(GlcNAc)_2$	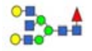
910.0450(3+)	2728.1193	2728.1188	0	756.3999	**[NA:144-150]**<br>(K)HS^{146}NGTIK(S)	1971.7189	$Gal_2(GlcNAc)_3Man_3Fuc(GlcNAc)_2$	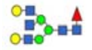
807.3416(3+)	2420.0092	2420.0081	0	756.3999	**[NA:144-150]**<br>(K)HS^{146}NGTIK(D)	1663.6082	$Gal(GlcNAc)_3Man_3(GlcNAc)_2$	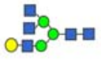
861.3593(3+)	2582.0622	2582.0609	1	756.3999	**[NA:144-150]**<br>(K)HS^{146}NGTIK(D)	1825.6610	$Gal_2(GlcNAc)_3Man_3(GlcNAc)_2$	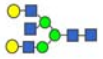
1108.9650(2+)	2216.9222	2216.9287	-3	756.3999	**[NA:144-150]**<br>(K)HS^{146}NGTIK(D)	1460.5288	$Gal(GlcNAc)_2Man_3(GlcNAc)_2$	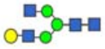
1210.5081(2+)	2420.0084	2420.0081	0	756.3999	**[NA:144-150]**<br>(K)HS^{146}NGTIK(S)	1663.6082	$Gal(GlcNAc)_3Man_3(GlcNAc)_2$	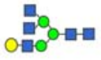
1291.5347(2+)	2582.0616	2582.0609	0	756.3999	**[NA:144-150]**<br>(K)HS^{146}NGTIK(D)	1825.6610	$Gal_2(GlcNAc)_3Man_3(GlcNAc)_2$	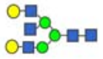
1210.5081(2+)	2420.0084	2420.0081	0	756.3999	**[NA:144-150]**<br>(K)HS^{146}NGTIK(S)	1663.6082	$Gal(GlcNAc)_3Man_3(GlcNAc)_2$	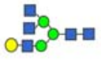
1283.5375(2+)	2566.0672	2566.0660	0	756.3999	**[NA:144-150]**<br>(K)HS^{146}NGTIK(S)	1809.6661	$Gal(GlcNAc)_3Man_3Fuc(GlcNAc)_2$	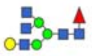
807.3416(3+)	2420.0091	2420.0081	0	756.3999	**[NA:144-150]**<br>(K)HS^{146}NGTIK(S)	1663.6082	$Gal(GlcNAc)_3Man_3(GlcNAc)_2$	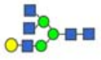
861.3593(3+)	2582.0622	2582.0609	1	756.3999	**[NA:144-150]**<br>(K)HS^{146}NGTIK(S)	1825.6610	$Gal_2(GlcNAc)_3Man_3(GlcNAc)_2$	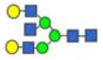
1210.5081(2+)	2420.0084	2420.0081	0	756.3999	**[NA:144-150]**<br>(K)HS^{146}NGTIK(S)	1663.6082	$Gal(GlcNAc)_3Man_3(GlcNAc)_2$	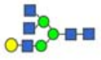
1291.5347(2+)	2582.0616	2582.0609	0	756.3999	**[NA:144-150]**<br>(K)HS^{146}NGTIK(S)	1825.6610	$Gal_2(GlcNAc)_3Man_3(GlcNAc)_2$	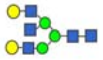
870.3667(3+)	2609.0844	2609.0830	1	1027.5279	**[NA:144-152]**<br>(K)HS^{146}NGTIKDR(S)	1581.5551	$Gal(GlcNAc)Man_5(GlcNAc)_2$	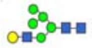
919.0525(3+)	2755.1418	2755.1409	0	1027.5279	**[NA:144-152]**<br>(K)HS^{146}NGTIKDR(S)	1727.6130	$Gal(GlcNAc)Man_5Fuc(GlcNAc)_2$	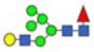

946.4038(3+)	2837.1958	2837.1940	1	1027.5279	**[NA:144-152]** (K)HS^{146}NGTIKDR(S)	1809.6661	Gal(GlcNAc)$_3$Man$_3$Fuc(GlcNAc)$_2$	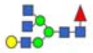
1000.4215(3+)	2999.2489	2999.2468	1	1027.5279	**[NA:144-152]** (K)HS^{146}NGTIKDR(S)	1971.7189	Gal$_2$(GlcNAc)$_3$Man$_3$Fuc(GlcNAc)$_2$	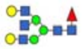
884.0422(3+)	2650.1109	2650.1095	1	1027.5279	**[NA:144-152]** (K)HS^{146}NGTIKDR(S)	1622.5816	Gal$_2$(GlcNAc)$_2$Man$_3$(GlcNAc)$_2$	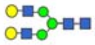
897.7177(3+)	2691.1374	2691.1361	1	1027.5279	**[NA:144-152]** (K)HS^{146}NGTIKDR(S)	1663.6082	Gal(GlcNAc)$_3$Man$_3$(GlcNAc)$_2$	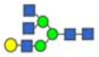
951.7355(3+)	2853.1908	2853.1889	1	1027.5279	**[NA:144-152]** (K)HS^{146}NGTIKDR(S)	1825.6610	Gal$_2$(GlcNAc)$_3$Man$_3$(GlcNAc)$_2$	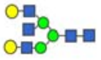
1046.0811(3+)	3136.2276	3136.2251	1	1027.5279	**[NA:144-152]** (K)HS^{146}NGTIKDR(S)	2108.6972	Gal$_2$(SO$_3$)(GlcNAc)$_4$Man$_3$(GlcNAc)$_2$	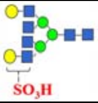
1100.0981(3+)	3298.2786	3298.2779	0	1027.5279	**[NA:144-152]** (K)HS^{146}NGTIKDR(S)	2270.7500	Gal$_3$(SO$_3$)(GlcNAc)$_4$Man$_3$(GlcNAc)$_2$	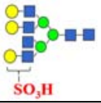
1148.7847(3+)	3444.3385	3444.3358	1	1027.5279	**[NA:144-152]** (K)HS^{146}NGTIKDR(S)	2416.8079	Gal$_3$(SO$_3$)(GlcNAc)$_4$Man$_3$Fuc(GlcNAc)$_2$	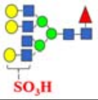

Table S3. Identification of glycopeptides of influenza proteins in the vaccine of NIBRG-121xp by LTQ-FT MS/MS analyses of a tryptic digest followed by proteinase K cleavage

Glycopeptide m/z (charge)	Meas. MH+	Calc. MH+	ppm	Peptide MH+	Peptide sequence [protein: residue position]	Glycan mass	Glycan composition	Glycan structure
824.6848(3+)	2472.0388	2472.0380	0	703.3985	**[HA: 37-42]** (V)LEK^{40}NVT(V)	1768.6395	Gal$_2$(GlcNAc)$_2$Man$_3$Fuc(GlcNAc)$_2$	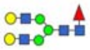
892.3779(3+)	2675.1181	2675.1174	0	703.3985	**[HA: 37-42]** (V)LEK^{40}NVT(V)	1971.7189	Gal$_2$(GlcNAc)$_3$Man$_3$Fuc(GlcNAc)$_2$	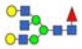
946.3956(3+)	2837.1712	2837.1702	0	703.3985	**[HA: 37-42]** (V)LEK^{40}NVT(V)	2133.7717	Gal$_3$(GlcNAc)$_3$Man$_3$Fuc(GlcNAc)$_2$	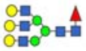
1014.0887(3+)	3040.2505	3040.2496	0	703.3985	**[HA: 37-42]** (V)LEK^{40}NVT(V)	2336.8511	Gal$_3$(GlcNAc)$_4$Man$_3$Fuc(GlcNAc)$_2$	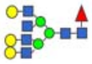
1068.1061(3+)	3202.3027	3202.3024	0	703.3985	**[HA: 37-42]** (V)LEK^{40}NVT(V)	2498.9039	Gal$_4$(GlcNAc)$_4$Man$_3$Fuc(GlcNAc)$_2$	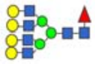
1053.9600(2+)	2106.9122	2106.9058	3	703.3985	**[HA: 37-42]** (V)LEK^{40}NVT(V)	1403.5073	Gal(GlcNAc)Man$_3$Fuc(GlcNAc)$_2$	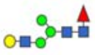
1134.9865(2+)	2268.9652	2268.9586	3	703.3985	**[HA: 37-42]** (V)LEK^{40}NVT(V)	1565.5601	(GlcNAc)Man$_5$Fuc(GlcNAc)$_2$	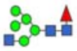
1142.9827(2+)	2284.9576	2284.9536	2	703.3985	**[HA: 37-42]** (V)LEK^{40}NVT(V)	1581.5551	Gal(GlcNAc)Man$_5$(GlcNAc)$_2$	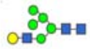
1216.0100(2+)	2431.0122	2431.0115	-4	703.3985	**[HA: 37-42]** (V)LEK^{40}NVT(V)	1727.6130	Gal(GlcNAc)Man$_5$Fuc(GlcNAc)$_2$	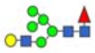
1155.4955(2+)	2309.9832	2309.9852	-1	703.3985	**[HA: 37-42]** (V)LEK^{40}NVT(V)	1606.5867	Gal(GlcNAc)$_2$Man$_3$Fuc(GlcNAc)$_2$	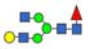
1236.5234(2+)	2472.0390	2472.0380	0	703.3985	**[HA: 37-42]** (V)LEK^{40}NVT(V)	1768.6395	Gal$_2$(GlcNAc)$_2$Man$_3$Fuc(GlcNAc)$_2$	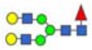
1309.5519(2+)	2618.0960	2618.0959	0	703.3985	**[HA: 37-42]** (V)LEK^{40}NVT(V)	1914.6974	Gal$_2$Fuc(GlcNAc)$_2$Man$_3$Fuc(GlcNAc)$_2$	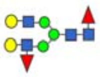
1338.0631(2+)	2675.1184	2675.1174	0	703.3985	**[HA: 37-42]** (V)LEK^{40}NVT(V)	1971.7189	Gal$_2$(GlcNAc)$_3$Man$_3$Fuc(GlcNAc)$_2$	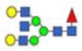
1411.0915(2+)	2821.1752	2821.1753	0	703.3985	**[HA: 37-42]** (V)LEK^{40}NVT(V)	2117.7768	Gal$_2$Fuc(GlcNAc)$_3$Man$_3$Fuc(GlcNAc)$_2$	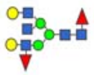
1346.0600(2+)	2691.1122	2691.1123	0	703.3985	**[HA: 37-42]** (V)LEK^{40}NVT(V)	1987.7138	Gal$_3$(GlcNAc)$_3$Man$_3$(GlcNAc)$_2$	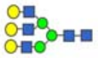

1419.0900(2+)	2837.1722	2837.1702	1	703.3985	**[HA: 37-42]** (V)LEK^{40}NVT(V)	2133.7717	Gal$_3$(GlcNAc)$_3$Man$_3$Fuc(GlcNAc)$_2$	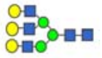
1492.1809(2+)	2983.2282	2983.2281	0	703.3985	**[HA: 37-42]** (V)LEK^{40}NVT(V)	2279.8296	Gal$_3$Fuc(GlcNAc)$_3$Man$_3$Fuc(GlcNAc)$_2$	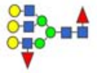
1520.6287(2+)	3040.2496	3040.2496	0	703.3985	**[HA: 37-42]** (V)LEK^{40}NVT(V)	2336.8511	Gal$_3$(GlcNAc)$_4$Man$_3$Fuc(GlcNAc)$_2$	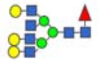
1601.6550(2+)	3202.3022	3202.3024	0	703.3985	**[HA: 37-42]** (V)LEK^{40}NVT(V)	2498.9039	Gal$_4$(GlcNAc)$_4$Man$_3$Fuc(GlcNAc)$_2$	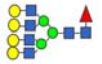
1179.9809(2+)	2358.9540	2358.9539	0	590.3144	**[HA: 38-42]** (L)EK^{40}NVT(V)	1768.6395	Gal$_2$(GlcNAc)$_2$Man$_3$Fuc(GlcNAc)$_2$	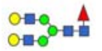
1281.5206(2+)	2562.0334	2562.0333	0	590.3144	**[HA: 38-42]** (L)EK^{40}NVT(V)	1971.7189	Gal$_2$(GlcNAc)$_3$Man$_3$Fuc(GlcNAc)$_2$	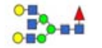
855.3097(2+)	1709.6116	1709.6118	0	493.1889	**[HA: 102-106]** (S)SD^{104}NGT(C)	1216.4229	Man$_5$(GlcNAc)$_2$	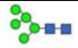
936.3366(2+)	1871.6654	1871.6646	0	493.1889	**[HA: 102-106]** (S)SD^{104}NGT(C)	1378.4757	Man$_6$(GlcNAc)$_2$	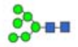
1037.8755(2+)	2074.7432	2074.7440	0	493.1889	**[HA: 102-106]** (S)SD^{104}NGT(C)	1581.5551	Gal(GlcNAc)Man$_5$(GlcNAc)$_2$	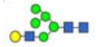
1058.3886(2+)	2115.7694	2115.7705	-1	493.1889	**[HA: 102-106]** (S)SD^{104}NGT(C)	1622.5816	Gal$_2$(GlcNAc)$_2$Man$_3$(GlcNAc)$_2$	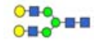
773.6229(3+)	2318.8531	2318.8499	1	493.1889	**[HA: 102-106]** (S)SD^{104}NGT(C)	1825.6610	Gal$_2$(GlcNAc)$_3$Man$_3$(GlcNAc)$_2$	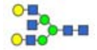
817.8130(2+)	1634.6182	1634.6161	1	418.1932	**[HA: 135-138]** (F) P^{136}NTS (S)	1216.4229	Man$_5$(GlcNAc)$_2$	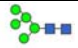
1004.9172(2+)	2008.8266	2008.8267	0	954.4567	**[HA: 131-138]** (R)FEIFP^{136}NTS(S)	1054.3700	Man$_4$(GlcNAc)$_2$	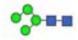
1085.9435(2+)	2170.8792	2170.8796	0	954.4567	**[HA: 131-138]** (R)FEIFP^{136}NTS(S)	1216.4229	Man$_5$(GlcNAc)$_2$	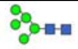
1166.9691(2+)	2332.9304	2332.9324	-1	954.4567	**[HA: 131-138]** (R)FEIFP^{136}NTS(S)	1378.4757	Man$_6$(GlcNAc)$_2$	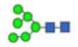
1247.9958(2+)	2494.9838	2494.9852	-1	954.4567	**[HA: 131-138]** (R)FEIFP^{136}NTS(S)	1540.5285	Man$_7$(GlcNAc)$_2$	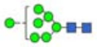
1268.5100(2+)	2536.0122	2536.0118	0	954.4567	**[HA: 131-138]** (R)FEIFP^{136}NTS(S)	1581.5551	Gal(GlcNAc)Man$_5$(GlcNAc)$_2$	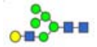
1370.0502(2+)	2739.0926	2739.0911	1	954.4567	**[HA: 131-138]** (R)FEIFP^{136}NTS(S)	1784.6344	(GlcNAc)$_2$Man$_6$(GlcNAc)$_2$	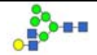
1451.0770(2+)	2901.1462	2901.1440	1	954.4567	**[HA: 131-138]** (R)FEIFP^{136}NTS(S)	1946.6873	(GlcNAc)$_2$Man$_7$(GlcNAc)$_2$	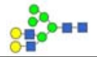
1341.5406(2+)	2682.0734	2682.0697	1	954.4567	**[HA: 131-138]** (R)FEIFP^{136}NTS(S)	1727.6130	Gal(GlcNAc)Man$_5$Fuc(GlcNAc)$_2$	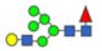
1362.0513(2+)	2723.0948	2723.0963	-1	954.4567	**[HA: 131-138]** (R)FEIFP^{136}NTS(S)	1768.6396	Gal$_2$(GlcNAc)$_2$Man$_3$Fuc(GlcNAc)$_2$	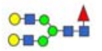
1443.0785(2+)	2885.1492	2885.1491	0	954.4567	**[HA: 131-138]** (R)FEIFP^{136}NTS(S)	1930.6924	Fuc(GlcNAc)$_2$Man$_5$(GlcNAc)$_2$Gal	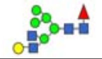
1382.5689(2+)	2764.1300	2764.1228	2	954.4567	**[HA: 131-138]** (R)FEIFP^{136}NTS(S)	1809.6661	Gal(GlcNAc)$_3$Man$_3$Fuc(GlcNAc)$_2$	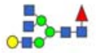
1463.5925(2+)	2926.1772	2926.1756	1	954.4567	**[HA: 131-138]** (R)FEIFP^{136}NTS(S)	1971.7189	Gal$_2$(GlcNAc)$_3$Man$_3$Fuc(GlcNAc)$_2$	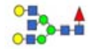
1536.6211(2+)	3072.2344	3072.2335	0	954.4567	**[HA: 131-138]** (R)FEIFP^{136}NTS(S)	2117.7768	Gal$_2$Fuc(GlcNAc)$_3$Man$_3$Fuc(GlcNAc)$_2$	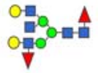
1609.6532(2+)	3218.2986	3218.2914	2	954.4567	**[HA: 131-138]** (R)FEIFP^{136}NTS(S)	2263.8347	Gal$_2$Fuc$_2$(GlcNAc)$_3$Man$_3$Fuc(GlcNAc)$_2$	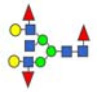
948.6989(3+)	2844.0810	2844.0796	1	954.4567	**[HA: 131-138]** (R)FEIFP^{136}NTS(S)	1889.6229	Fuc(GlcNAc)$_2$Man$_3$(GlcNAc)$_3$Gal(SO_3)	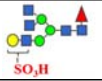
1002.7166(3+)	3006.1342	3006.1324	1	954.4567	**[HA: 131-138]** (R)FEIFP^{136}NTS(S)	2051.6757	Gal$_2$(SO_3)(GlcNAc)$_3$Man$_3$Fuc(GlcNAc)$_2$	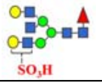

1056.7340(3+)	3168.1864	3168.1852	0	954.4567	**[HA: 131-138]** (R)FEIFP^{136}NTS(S)	2213.7285	Gal$_3$(SO$_3$)(GlcNAc)$_3$Man$_3$Fuc(GlcNAc)$_2$	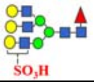
1124.4274(3+)	3371.2665	3371.2646	1	954.4567	**[HA: 131-138]** (R)FEIFP^{136}NTS(S)	2416.8079	Gal$_3$(SO$_3$)(GlcNAc)$_4$Man$_3$Fuc(GlcNAc)$_2$	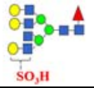
1173.1134(3+)	3517.3246	3517.3225	1	954.4567	**[HA: 131-138]** (R)FEIFP^{136}NTS(S)	2562.8658	Gal$_3$(SO$_3$)Fuc(GlcNAc)$_4$Man$_3$Fuc(GlcNAc)$_2$	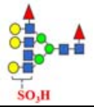
1221.8000(3+)	3663.3844	3663.3804	1	954.4567	**[HA: 131-138]** (R)FEIFP^{136}NTS(S)	2708.9237	Gal$_3$(SO$_3$)Fuc(GlcNAc)$_4$Man$_3$Fuc$_2$(GlcNAc)$_2$	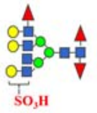
1584.6016(2+)	3168.1954	3168.1852	3	954.4567	**[HA: 131-138]** (R)FEIFP^{136}NTS(S)	2213.7285	Gal$_3$(SO$_3$)(GlcNAc)$_3$Man$_3$Fuc(GlcNAc)$_2$	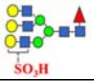
1686.1373(2+)	3371.2668	3371.2646	1	954.4567	**[HA: 131-138]** (R)FEIFP^{136}NTS(S)	2416.8079	Gal$_3$(SO$_3$)(GlcNAc)$_4$Man$_3$Fuc(GlcNAc)$_2$	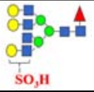
1759.1687(2+)	3517.3296	3517.3225	2	954.4567	**[HA: 131-138]** (R)FEIFP^{136}NTS(S)	2562.8658	Fuc(GlcNAc)$_2$Man$_3$Fuc(GlcNAc)$_4$Gal$_3$(SO$_3$)	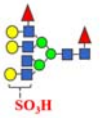
1188.4504(2+)	2375.8930.	2375.8913	1	1159.4684	**[HA: 286-295]** (S)DTPVHDC^{293}NTT(C)	1216.4229	Man$_5$(GlcNAc)$_2$	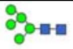
1464.5581(2+)	2928.1084	2928.1079	0	1159.4684	**[HA: 286-295]** (S)DTPVHDC^{293}NTT(C)	1768.6395	Gal$_2$(GlcNAc)$_2$Man$_3$Fuc(GlcNAc)$_2$	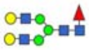
1566.0980(2+)	3131.1882	3131.1873	0	1159.4684	**[HA: 286-295]** (S)DTPVHDC^{293}NTT(C)	1971.7189	Gal$_2$(GlcNAc)$_3$Man$_3$Fuc(GlcNAc)$_2$	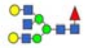
1647.1244(2+)	3293.2410	3293.2401	0	1159.4684	**[HA: 286-295]** (S)DTPVHDC^{293}NTT(C)	2133.7717	Gal$_3$(GlcNAc)$_3$Man$_3$Fuc(GlcNAc)$_2$	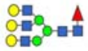
976.7081(3+)	2928.1087	2928.1079	0	1159.4684	**[HA: 286-295]** (S)DTPVHDC^{293}NTT(C)	1768.6395	Gal$_2$(GlcNAc)$_2$Man$_3$Fuc(GlcNAc)$_2$	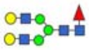
1044.4010(3+)	3131.1874	3131.1873	0	1159.4684	**[HA: 286-295]** (S)DTPVHDC^{293}NTT(C)	1971.7189	Gal$_2$(GlcNAc)$_3$Man$_3$Fuc(GlcNAc)$_2$	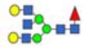
1098.4184(3+)	3293.2396	3293.2401	0	1159.4684	**[HA: 286-295]** (S)DTPVHDC^{293}NTT(C)	2133.7717	Gal$_3$(GlcNAc)$_3$Man$_3$Fuc(GlcNAc)$_2$	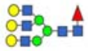
765.3145(2+)	1529.6212	1529.6211	0	475.2511	**[HA: 301-305]** (K)GAI^{304}NT(S)	1054.3700	Man$_4$(GlcNAc)$_2$	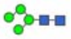
846.3411(2+)	1691.6744	1691.6740	0	475.2511	**[HA: 301-305]** (K)GAI^{304}NT(S)	1216.4229	Man$_5$(GlcNAc)$_2$	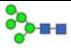
927.3681(2+)	1853.7284	1853.7268	1	475.2511	**[HA: 301-305]** (K)GAI^{304}NT(S)	1378.4757	Man$_6$(GlcNAc)$_2$	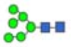
1008.3941(2+)	2015.7804	2015.7796	0	475.2511	**[HA: 301-305]** (K)GAI^{304}NT(S)	1540.5285	Man$_7$(GlcNAc)$_2$	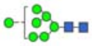
1089.4204(2+)	2177.8330	2177.8325	0	475.2511	**[HA: 301-305]** (K)GAI^{304}NT(S)	1702.5814	Man$_8$(GlcNAc)$_2$	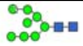
1184.9512(2+)	2368.8946	2368.8906	2	454.1932	**[HA: 498-502]** (K)^{498}NGTY(D)	1914.6974	Gal$_2$Fuc(GlcNAc)$_2$Man$_3$Fuc(GlcNAc)$_2$	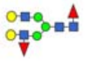
1187.4875(2+)	2373.9672	2373.9648	1	605.3253	**[HA: 495-500]** (E)SVK^{498}NGT(Y)	1768.6395	Gal$_2$(GlcNAc)$_2$Man$_3$Fuc(GlcNAc)$_2$	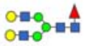
1260.5159(2+)	2520.0240	2520.0228	1	605.3253	**[HA: 495-500]** (E)SVK^{498}NGT(Y)	1914.6974	Gal$_2$Fuc(GlcNAc)$_2$Man$_3$Fuc(GlcNAc)$_2$	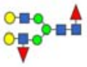
1289.0271(2+)	2577.0464	2577.0442	1	605.3253	**[HA: 495-500]** (E)SVK^{498}NGT(Y)	1971.7189	Gal$_2$(GlcNAc)$_3$Man$_3$Fuc(GlcNAc)$_2$	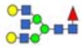
1370.0560(2+)	2739.1042	2739.0970	3	605.3253	**[HA: 495-500]** (E)SVK^{498}NGT(Y)	2133.7717	Gal$_3$(GlcNAc)$_3$Man$_3$Fuc(GlcNAc)$_2$	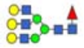
791.9939(3+)	2373.9661	2373.9648	0	605.3253	**[HA: 495-500]** (E)SVK^{498}NGT(Y)	1768.6395	Gal$_2$(GlcNAc)$_2$Man$_3$Fuc(GlcNAc)$_2$	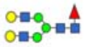

840.6802(3+)	2520.0249	2520.0228	1	605.3253	**[HA: 495-500]**<br>(E)SVK^{498}NGT(Y)	1914.6974	Gal$_2$Fuc(GlcNAc)$_2$Man$_3$Fuc(GlcNAc)$_2$	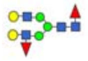
1245.0208(2+)	2489.0338	2489.0335	0	1085.5262	**[HA: 497-505]**<br>(V)K^{498}N GTYDYPK(Y)	1403.5073	Gal(GlcNAc)Man$_3$Fuc(GlcNAc)$_2$	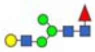
1346.5615(2+)	2692.1152	2692.1129	1	1085.5262	**[HA: 497-505]**<br>(V)K^{498}N GTYDYPK(Y)	1606.5867	Gal(GlcNAc)$_2$Man$_3$Fuc(GlcNAc)$_2$	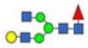
1427.5878(2+)	2854.1678	2854.1657	1	1085.5262	**[HA: 497-505]**<br>(V)K^{498}N GTYDYPK(Y)	1768.6395	Gal$_2$(GlcNAc)$_2$Man$_3$Fuc(GlcNAc)$_2$	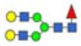
1529.1301(2+)	3057.2524	3057.2451	3	1085.5262	**[HA: 497-505]**<br>(V)K^{498}N GTYDYPK(Y)	1971.7189	Gal$_2$(GlcNAc)$_3$Man$_3$Fuc(GlcNAc)$_2$	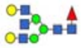
1610.1540(2+)	3219.3002	3219.2979	1	1085.5262	**[HA: 497-505]**<br>(V)K^{498}N GTYDYPK(Y)	2133.7717	Gal$_3$(GlcNAc)$_3$Man$_3$Fuc(GlcNAc)$_2$	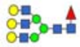
952.0580(3+)	2854.1584	2854.1657	-3	1085.5262	**[HA: 497-505]**<br>(V)K^{498}N GTYDYPK(Y)	1768.6395	Gal$_2$(GlcNAc)$_2$Man$_3$Fuc(GlcNAc)$_2$	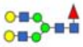
1019.7505(3+)	3057.2359	3057.2451	-3	1085.5262	**[HA: 497-505]**<br>(V)K^{498}N GTYDYPK(Y)	1971.7189	Gal$_2$(GlcNAc)$_3$Man$_3$Fuc(GlcNAc)$_2$	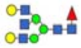
1073.7710(3+)	3219.2974	3219.2979	0	1085.5262	**[HA: 497-505]**<br>(V)K^{498}N GTYDYPK(Y)	2133.7717	Gal$_3$(GlcNAc)$_3$Man$_3$Fuc(GlcNAc)$_2$	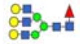
1151.4622(2+)	2301.9166	2301.9161	0	1085.4932	**[NA:48-56]**<br>(E)TC^{50}NQSVITY(E)	1216.4229	Man$_5$(GlcNAc)$_2$	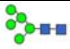
767.9776(3+)	2301.9172	2301.9161	1	1085.4932	**[NA:48-56]**<br>(E)TC^{50}NQSVITY(E)	1216.4229	Man$_5$(GlcNAc)$_2$	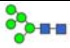
821.9950(3+)	2463.9694	2463.9689	0	1085.4932	**[NA:48-56]**<br>(E)TC^{50}NQSVITY(E)	1378.4757	Man$_6$(GlcNAc)$_2$	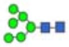
876.0127(3+)	2626.0224	2626.0217	0	1085.4932	**[NA:48-56]**<br>(E)TC^{50}NQSVITY(E)	1540.5285	Man$_7$(GlcNAc)$_2$	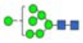
903.3618(3+)	2708.0698	2708.0748	-2	1085.4932	**[NA:48-56]**<br>(E)TC^{50}NQSVITY(E)	1622.5816	Gal$_2$(GlcNAc)$_2$Man$_3$(GlcNAc)$_2$	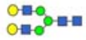
952.0510(3+)	2854.1374	2845.1327	2	1085.4932	**[NA:48-56]**<br>(E)TC^{50}NQSVITY(E)	1768.6395	Gal$_2$(GlcNAc)$_2$Man$_3$Fuc(GlcNAc)$_2$	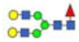
1040.3744(2+)	2079.7410	2079.7381	1	539.2096	**[NA: 56-59]**<br>(T)YE^{58}NN(T)	1540.5285	Man$_7$(GlcNAc)$_2$	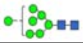
1121.4021(2+)	2241.7964	2241.7910	2	539.2096	**[NA: 56-59]**<br>(T)YE^{58}NN(T)	1702.5814	Man$_8$(GlcNAc)$_2$	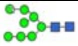
1202.4276(2+)	2403.8474	2403.8438	2	539.2096	**[NA: 56-59]**<br>(T)YE^{58}NN(T)	1864.6342	Man$_9$(GlcNAc)$_2$	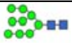
987.3929(2+)	1973.7780	1973.7843	-3	595.3086	**[NA: 66-70]**<br>(T)YV^{68}NIS(N)	1378.4757	Man$_6$(GlcNAc)$_2$	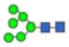
1068.4235(2+)	2135.8392	2135.8371	1	595.3086	**[NA: 66-70]**<br>(T)YV^{68}NIS(N)	1540.5285	Man$_7$(GlcNAc)$_2$	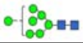
1149.4500(2+)	2297.8922	2297.8900	1	595.3086	**[NA: 66-70]**<br>(T)YV^{68}NIS(N)	1702.5814	Man$_8$(GlcNAc)$_2$	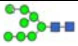
1230.4764(2+)	2459.9450	2459.9428	1	595.3086	**[NA: 66-70]**<br>(T)YV^{68}NIS(N)	1864.6342	Man$_9$(GlcNAc)$_2$	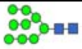
1089.9202(2+)	2178.8326	2178.8291	2	515.2209	**[NA:144-148]**<br>(K)HS^{146}N GT(I)<br>20120117-02	1663.6082	Gal(GlcNAc)$_3$Man$_3$(GlcNAc)$_2$	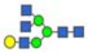
1170.9456(2+)	2340.8834	2340.8819	1	515.2209	**[NA:144-148]**<br>(K)HS^{146}N GT(I)	1825.6610	Gal$_2$(GlcNAc)$_3$Man$_3$(GlcNAc)$_2$	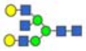
780.9637(3+)	2340.8755	2340.8819	3	515.2209	**[NA:144-148]**<br>(K)HS^{146}N GT(I)<br>20120117-02	1825.6610	Gal$_2$(GlcNAc)$_3$Man$_3$(GlcNAc)$_2$	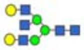
792.2897(3+)	2374.8535	2374.8518	1	752.2702	**[NA: 230-235]**<br>(S)ECACV^{235}N(GS)	1622.5816	Gal$_2$(GlcNAc)$_2$Man$_3$(GlcNAc)$_2$	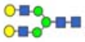
1030.3677(2+)	20597276	2059.7265	1	437.1449	**[NA, 235-238]**<br>(V)^{235}NGSC(F)	1622.5816	Gal$_2$(GlcNAc)$_2$Man$_3$(GlcNAc)$_2$	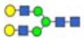
786.9951(3+)	2358.9696	2358.9804	4	736.3988	**[NA:386-391]**<br>(N)^{386}N FSIKQ(D)	1622.5816	Gal$_2$(GlcNAc)$_2$Man$_3$(GlcNAc)$_2$	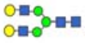

Table S4. Identification of glycopeptides of influenza proteins in the vaccine of NYMC-X181A by LTQ-FT MS/MS analyses of a tryptic digest followed by proteinase K cleavage

Glycopeptide m/z (charge)	Meas.Mass	Calc. Mass MH+	ppm	Peptide MH+	Peptide sequence [protein: residue position]	Glycan mass	Glycan composition	Glycan structure
1056.3975(2+)	2111.7872	2111.7868	0	651.2580	**[HA, 27-32]**<br>(A)N^{28}NSTDT(V)	1460.5288	Gal(GlcNAc)$_2$Man$_3$(GlcNAc)$_2$	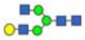

824.6852(3+)	2472.03995	2472.0380	1	703.3985	**[HA: 37-42]**<br>(V)LEK^{40}NVT(V)	1768.6395	Gal$_2$(GlcNAc)$_2$Man$_3$Fuc(GlcNAc)$_2$	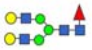
892.3776(3+)	2675.1172	2675.1174	0	703.3985	**[HA: 37-42]**<br>(V)LEK^{40}NVT(V)	1971.7189	Gal$_2$(GlcNAc)$_3$Man$_3$Fuc(GlcNAc)$_2$	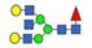
946.3955(3+)	2837.1709	2837.1702	0	703.3985	**[HA: 37-42]**<br>(V)LEK^{40}NVT(V)	2133.7717	Gal$_3$(GlcNAc)$_3$Man$_3$Fuc(GlcNAc)$_2$	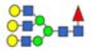
1014.0891(3+)	3040.2516	3040.2496	1	703.3985	**[HA: 37-42]**<br>(V)LEK^{40}NVT(V)	2336.8511	Gal$_3$(GlcNAc)$_4$Man$_3$Fuc(GlcNAc)$_2$	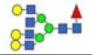
1053.9598(2+)	2106.9118	2106.9058	3	703.3985	**[HA: 37-42]**<br>(V)LEK^{40}NVT(V)	1403.5073	Gal(GlcNAc)Man$_3$Fuc(GlcNAc)$_2$	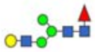
1134.9863(2+)	2268.9648	2268.9586	3	703.3985	**[HA: 37-42]**<br>(V)LEK^{40}NVT(V)	1565.5601	(GlcNAc)Man$_5$Fuc(GlcNAc)$_2$	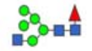
1126.9888(2+)	2252.9698	2252.9637	3	703.3985	**[HA: 37-42]**<br>(V)LEK^{40}NVT(V)	1549.5652	Fuc(GlcNAc)$_2$Man$_3$Fuc(GlcNAc)Gal	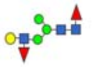
1142.9825(2+)	2284.9572	2284.9536	1	703.3985	**[HA: 37-42]**<br>(V)LEK^{40}NVT(V)	1581.5551	Gal(GlcNAc)Man$_5$(GlcNAc)$_2$	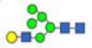
1163.4942(2+)	2325.9806	2325.9801	0	703.3985	**[HA: 37-42]**<br>(V)LEK^{40}NVT(V)	1622.5816	Gal$_2$(GlcNAc)$_2$Man$_3$(GlcNAc)$_2$	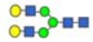
1236.5225(2+)	2472.0372	2472.0380	0	703.3985	**[HA: 37-42]**<br>(V)LEK^{40}NVT(V)	1768.6395	Gal$_2$(GlcNAc)$_2$Man$_3$Fuc(GlcNAc)$_2$	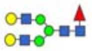
1309.5527(2+)	2618.0976	2618.0959	0	703.3985	**[HA: 37-42]**<br>(V)LEK^{40}NVT(V)	1914.6974	Gal$_2$Fuc(GlcNAc)$_2$Man$_3$Fuc(GlcNAc)$_2$	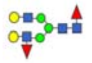
1338.0631(2+)	2675.1184	2675.1174	0	703.3985	**[HA: 37-42]**<br>(V)LEK^{40}NVT(V)	1971.7189	Gal$_2$(GlcNAc)$_3$Man$_3$Fuc(GlcNAc)$_2$	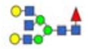
1382.5834(2+)	2764.1590	2764.1538	2	703.3985	**[HA: 37-42]**<br>(V)LEK^{40}NVT(V)	2060.7553	Gal$_2$Fuc$_2$(GlcNAc)$_2$Man$_3$Fuc(GlcNAc)$_2$	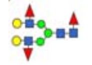
1411.0911(2+)	2821.1744	2821.1753	0	703.3985	**[HA: 37-42]**<br>(V)LEK^{40}NVT(V)	2117.7768	Gal$_2$Fuc(GlcNAc)$_3$Man$_3$Fuc(GlcNAc)$_2$	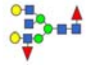
1419.0890(2+)	2837.1702	2837.1702	0	703.3985	**[HA: 37-42]**<br>(V)LEK^{40}NVT(V)	2133.7717	Gal$_3$(GlcNAc)$_3$Man$_3$Fuc(GlcNAc)$_2$	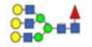
1492.1199(2+)	2983.2320	2983.2281	1	703.3985	**[HA: 37-42]**<br>(V)LEK^{40}NVT(V)	2279.8296	Gal$_3$Fuc(GlcNAc)$_3$Man$_3$Fuc(GlcNAc)$_2$	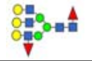
1520.6287(2+)	3040.2496	3040.2496	0	703.3985	**[HA: 37-42]**<br>(V)LEK^{40}NVT(V)	2336.8511	Gal$_3$(GlcNAc)$_4$Man$_3$Fuc(GlcNAc)$_2$	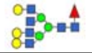
1159.4649(2+)	2317.9220	2317.9274	2	590.3144	**[HA:38-42]**<br>(L)EK^{40}NVT(V)	1727.6130	Gal(GlcNAc)Man$_5$Fuc(GlcNAc)$_2$	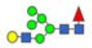
1179.9835(2+)	2358.9592	2358.9539	2	590.3144	**[HA:38-42]**<br>(L)EK^{40}NVT(V)	1768.6395	Gal$_2$(GlcNAc)$_2$Man$_3$Fuc(GlcNAc)$_2$	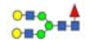
1281.5228(2+)	2562.0378	2562.0333	2	590.3144	**[HA:38-42]**<br>(L)EK^{40}NVT(V)	1971.7189	Gal$_2$(GlcNAc)$_3$Man$_3$Fuc(GlcNAc)$_2$	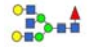
855.3102(2+)	1709.6126	1709.6118	0	493.1889	**[HA:102-104]**<br>(S)SD^{104}N(GT)	1216.4229	Man$_5$(GlcNAc)$_2$	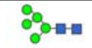
936.3340(2+)	1871.6602	1871.6646	2	493.1889	**[HA:102-104]**<br>(S)SD^{104}N(GT)	1378.4757	Man$_6$(GlcNAc)$_2$	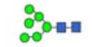
1037.8749(2+)	2074.7420	2074.7440	1	493.1889	**[HA: 102-106]**<br>(S)SD^{104}NGT(C)	1581.5551	Gal(GlcNAc)Man$_5$(GlcNAc)$_2$	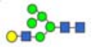
765.3145(2+)	1529.6212	1529.6211	0	475.2511	**[HA, 301-305}**<br>(K)GAINT(S)	1054.3700	Man$_4$(GlcNAc)$_2$	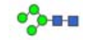
846.3411(2+)	1691.6744	1691.6740	0	475.2511	**[HA: 301-305]**<br>(K)GAI^{304}NT(S)	1216.4229	Man$_5$(GlcNAc)$_2$	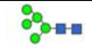
927.3676(2+)	1853.7274	1853.7268	0	475.2511	**[HA: 301-305]**<br>(K)GAI^{304}NT(S)	1378.4757	Man$_6$(GlcNAc)$_2$	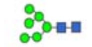
1008.3940(2+)	2015.7802	2015.7796	0	475.2511	**[HA: 301-305]**<br>(K)GAI^{304}NT(S)	1540.5285	Man$_7$(GlcNAc)$_2$	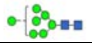
1089.4200(2+)	2177.8322	2177.8325	0	475.2511	**[HA: 301-305]**<br>(K)GAI^{304}NT(S)	1702.5814	Man$_8$(GlcNAc)$_2$	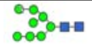
782.3127(2+)	1563.6176	1563.6154	1	347.1925	[HA:303-305]<br>(A)I^{304}NT(S)	1216.4229	Man$_5$(GlcNAc)$_2$	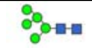
863.3389(2+)	1725.6700	1725.6682	1	347.1925	[HA:303-305]<br>(A)I^{304}NT(S)	1378.4757	Man$_6$(GlcNAc)$_2$	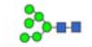
1187.4863(2+)	2373.9648	2373.9648	0	605.3253	**[HA, 495-500]**<br>(E)SVK^{498}NGT(Y)	1768.6395	Gal$_2$(GlcNAc)$_2$Man$_3$Fuc(GlcNAc)$_2$	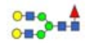

1289.0241(2+)	2577.0404	2577.0442	-1	605.3253	**[HA, 495-500]** (E)SVK^{498}NGT(Y)	1971.7189	Gal$_2$(GlcNAc)$_3$Man$_3$Fuc(GlcNAc)$_2$	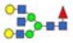
1370.0534(2+)	2739.0990	2739.0970	1	605.3253	**[HA, 495-500]** (E)SVK^{498}NGT(Y)	2133.7717	Gal$_3$(GlcNAc)$_3$Man$_3$Fuc(GlcNAc)$_2$	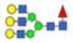
1040.3746(2+)	2079.7414	2079.7381	2	539.2096	**[NA: 56-59]** (T)YE^{58}NN(T)	1540.5285	Man$_7$(GlcNAc)$_2$	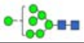
1121.4020(2+)	2241.7962	2241.7910	2	539.2096	**[NA: 56-59]** (T)YE^{58}NN(T)	1702.5814	Man$_8$(GlcNAc)$_2$	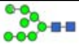
1202.4275(2+)	2403.8472	2403.8438	1	539.2096	**[NA: 56-59]** (T)YE^{58}NN(T)	1864.6342	Man$_9$(GlcNAc)$_2$	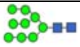
754.9554(3+)	2262.8506	2262.8389	5	640.2573	**[NA: 56-60]** (T)YE^{58}NNT(W)	1622.5816	Gal$_2$(GlcNAc)$_2$Man$_3$(GlcNAc)$_2$	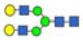
1068.4230(2+)	2135.8382	2135.8371	0	595.3086	**[NA, 66-70]** (T)YV^{68}NIS(N)	1540.5285	Man$_7$(GlcNAc)$_2$	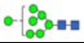
1149.4500(2+)	2297.8922	2297.8900	1	595.3086	**[NA, 66-70]** (T)YV^{68}NIS(N)	1702.5814	Man$_8$(GlcNAc)$_2$	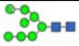
1230.4785(2+)	2459.9492	2459.9428	2	595.3086	**[NA, 66-70]** (T)YV^{68}NIS(N)	1864.6342	Man$_9$(GlcNAc)$_2$	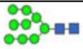
1013.4097(2+)	2025.8114	2025.8116	0	647.3359	**[NA, 67-72]** (Y)V^{68}NISNT(N)	1378.4757	Man$_6$(GlcNAc)$_2$	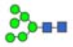
1094.4361(2+)	2187.8644	2187.8644	0	647.3359	**[NA, 67-72]** (Y)V^{68}NISNT(N)	1540.5285	Man$_7$(GlcNAc)$_2$	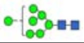
1175.4610(2+)	2349.9142	2349.9173	-1	647.3359	**[NA, 67-72]** (Y)V^{68}NISNT(N)	1702.5814	Man$_8$(GlcNAc)$_2$	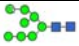
1256.4940(2+)	2511.9802	2511.9701	4	647.3359	**[NA, 67-72]** (Y)V^{68}NISNT(N)	1864.6342	Man$_9$(GlcNAc)$_2$	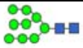
1089.9209(2+)	2178.8340	2178.8291	2	515.2209	**[NA, 144-148]** (K)HS^{146}NGT(I)	1663.6082	Gal(GlcNAc)$_3$Man$_3$(GlcNAc)$_2$	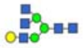
1170.9464(2+)	2340.8850	2340.8819	1	515.2209	**[NA, 144-148]** (K)HS^{146}NGT(I)	1825.6610	Gal$_2$(GlcNAc)$_3$Man$_3$(GlcNAc)$_2$	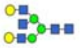
876.8247(2+)	1752.6416	1752.6362	3	536.2133	**[NA:233-237]** (A)CV^{235}NGS(C)	1216.4229	Man$_5$(GlcNAc)$_2$	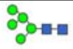
957.8495(2+)	1914.6912	1914.6890	1	536.2133	**[NA:233-237]** (A)CV^{235}NGS(C)	1378.4757	Man$_6$(GlcNAc)$_2$	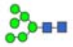
1038.8763(2+)	2076.7448	2076.7418	2	536.2133	**[NA:233-237]** (A)CV^{235}NGS(C)	1540.5285	Man$_7$(GlcNAc)$_2$	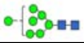
773.6129(3+)	2318.8231	2318.8256	-1	696.2440	**[NA:233-238]** (A)CV^{235}NGSC(F)	1622.5816	Gal$_2$(GlcNAc)$_2$Man$_3$(GlcNAc)$_2$	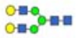
822.3019(3+)	2464.8900	2464.8835	3	696.2440	**[NA:233-238]** (A)CV^{235}NGSC(F)	1768.6395	Gal$_2$(GlcNAc)$_2$Man$_3$Fuc(GlcNAc)$_2$	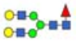
1030.3688(2+)	2059.7298	2059.7265	2	437.1449	[NA, 235-238] (V)^{235}NGSC(F)	1622.5816	Gal$_2$(GlcNAc)$_2$Man$_3$(GlcNAc)$_2$	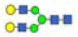

Table S5. Identification of glycopeptides of influenza proteins in the vaccine of NIBRG-121xp by LTQ-FT MS/MS analyses of the tryptic digest followed by chymotrypsin cleavage

m/z (charge)	Meas. MH+	Calc. MH+	ppm	Peptide MH$^+$	Peptide sequence [protein: residue position]	Glycan mass	Composition	Glycan structure
881.1272(4+)	3521.4853	3521.4861	0	2305.0632	**[HA: 129-147]** (F)ERFEIFP^{136}NTS SWPNHDSNK(G)	1216.4229	Man$_5$(GlcNAc)$_2$	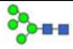
1030.4297(3+)	3089.2735	3089.2740	0	1872.8511	**[HA: 132-147]** (F)EIFP^{136}NTS SWPNHDSNK(G)	1216.4229	Man$_5$(GlcNAc)$_2$	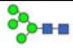
1084.4495(3+)	3251.3328	3251.3268	-2	1872.8511	**[HA: 132-147]** (F)EIFP^{136}NTS SWPNHDSNK(G)	1378.4757	Man$_6$(GlcNAc)$_2$	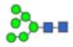
1152.1408(3+)	3454.4068	3454.4062	0	1872.8511	**[HA: 132-147]** (F)EIFP^{136}NTS SWPNHDSNK(G)	1581.5551	Gal(GlcNAc)Man$_5$(GlcNAc)$_2$	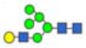
1219.8349(3+)	3657.4891	3657.4855	1	1872.8511	**[HA: 132-147]** (F)EIFP^{136}NTS SWPNHDSNK(G)	1784.6344	(GlcNAc)$_2$Man$_6$(GlcNAc)$_2$	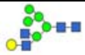
1273.8510(3+)	3819.5374	3819.5384	0	1872.8511	**[HA: 132-147]** (F)EIFP^{136}NTS SWPNHDSNK(G)	1946.6873	(GlcNAc)$_2$Man$_7$(GlcNAc)$_2$	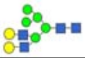
1228.1784(3+)	3682.5195	3682.5172	1	1872.8511	**[HA: 132-147]** (F)EIFP^{136}NTS SWPNHDSNK(G)	1809.6661	Gal(GlcNAc)$_3$Man$_3$Fuc(GlcNAc)$_2$	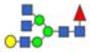
1282.1969(3+)	3844.5750	3844.5700	1	1872.8511	**[HA: 132-147]** (F)EIFP^{136}NTS SWPNHDSNK(G)	1971.7189	Fuc(GlcNAc)$_2$Man$_3$(GlcNAc)$_3$Gal$_2$	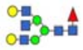
1079.4543(3+)	3236.3473	3236.3424	2	2019.9195	**[HA: 131-147]** (R)FEIFP^{136}NTSSWPNHDSNK(G)	1216.4229	Man$_5$(GlcNAc)$_2$	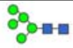
1201.1642(3+)	3601.4770	3601.4746	1	2019.9195	**[HA: 131-147]** (R)FEIFP^{136}NTSSWPNHDSNK(G)	1581.5551	Gal(GlcNAc)Man$_5$(GlcNAc)$_2$	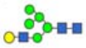
1331.2190(3+)	3991.6414	3991.6384	1	2019.9195	**[HA: 131-147]** (R)FEIFP^{136}NTSSWPNHDSNK(G)	1971.7189	Gal$_2$(GlcNAc)$_3$Man$_3$Fuc(GlcNAc)$_2$	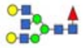

1268.2101(3+)	3802.6147	3802.6152	0	2586.1923	**[HA: 277-300]** (R) NAGSGIIISDTPVHDC^{293}NTT CQTPK (G)	1216.4229	Man$_{5}$(GlcNAc)$_{2}$	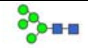
1322.2308(3+)	3964.6768	3964.6680	2	2586.1923	**[HA: 277-300]** (R) NAGSGIIISDTPVHDC^{293}NTT CQTPK (G)	1378.4757	Man$_{6}$(GlcNAc)$_{2}$	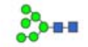
1389.9218(3+)	4167.7497	4167.7474	1	2586.1923	**[HA: 277-300]** (R) NAGSGIIISDTPVHDC^{293}NTT CQTPK (G)	1581.5551	Gal(GlcNAc)Man$_{5}$(GlcNAc)$_{2}$	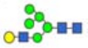
1438.6078(3+)	4313.8077	4313.8053	1	2586.1923	**[HA:277-300]** (R)NAGSGIIISDTPVHDC^{293}NTTCQTPK(G)	1727.6130	Gal(GlcNAc)Man$_{5}$Fuc(GlcNAc)$_{2}$	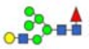
1452.2858(3+)	4354.8417	4354.8318	2	2586.1923	**[HA: 277-300]** (R) NAGSGIIISDTPVHDC^{293}NTT CQTPK (G)	1768.6395	Gal$_{2}$(GlcNAc)$_{2}$Man$_{3}$Fuc(GlcNAc)$_{2}$	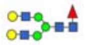
1519.9760(3+)	4557.9123	4557.9112	0	2586.1923	**[HA: 277-300]** (R) NAGSGIIISDTPVHDC^{293}NTT CQTPK (G)	1971.7189	Gal$_{2}$(GlcNAc)$_{3}$Man$_{3}$Fuc(GlcNAc)$_{2}$	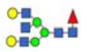
1079.8520(3+)	3237.5404	3237.5407	0	2021.1178	**[HA: 301-319]** (K)GAI^{304}NTSLPFQNIHPITIGK(C)	1216.4229	Man$_{5}$(GlcNAc)$_{2}$	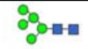
1133.8703(3+)	3399.5952	3399.5935	1	2021.1178	**[HA: 301-319]** (K)GAI^{304}NTSLPFQNIHPITIGK(C)	1378.4757	Man$_{6}$(GlcNAc)$_{2}$	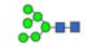
1187.8867(2+)	3561.6445	3561.6463	1	2021.1178	**[HA: 301-319]** (K)GAI^{304}NTSLPFQNIHPITIGK(C)	1540.5285	Man$_{7}$(GlcNAc)$_{2}$	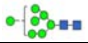
1241.9058(3+)	3723.7017	3723.6992	1	2021.1178	**[HA: 301-319]** (K)GAI^{304}NTSLPFQNIHPITIGK(C)	1702.5814	Man$_{8}$(GlcNAc)$_{2}$	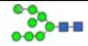
1096.4474(3+)	3287.3265	3287.3255	0	1664.7439	**[HA: 498-511]** (K)^{498}NGTYDYPKYSEEAK(L)	1622.5816	Gal$_{2}$(GlcNAc)$_{2}$Man$_{3}$(GlcNAc)$_{2}$	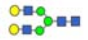
1145.1331(3+)	3433.3836	3433.3834	0	1664.7439	**[HA: 498-511]** (K)^{498}NGTYDYPKYSEEAK(L)	1768.6395	Gal$_{2}$(GlcNAc)$_{2}$Man$_{3}$Fuc(GlcNAc)$_{2}$	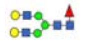
1193.8185(3+)	3579.4398	3579.4413	0	1664.7439	**[HA: 498-511]** (K)^{498}NGTYDYPKYSEEAK(L)	1914.6974	Gal$_{2}$Fuc(GlcNAc)$_{2}$Man$_{3}$Fuc(GlcNAc)$_{2}$	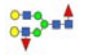
972.4111(4+)	3886.6209	3886.6176	1	2467.1154	**[NA:39-59]** (I)QLGNQNQIETC^{50}NQSVITYE^{58}NN(T)	1419.5022	(GlcNAc)Man$_{5}$(GlcNAc)$_{2}$	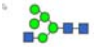
1023.1822(4+)	4089.7053	4089.6970	2	2467.1154	**[NA:39-59]** (I)QLGNQNQIETC^{50}NQSVITYE^{58}NN(T)	1622.5816	Gal$_{2}$(GlcNAc)$_{2}$Man$_{3}$(GlcNAc)$_{2}$	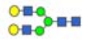
1063.6948(4+)	4251.7557	4251.7498	1	2467.1154	**[NA:39-59]** (I)QLGNQNQIETC^{50}NQSVITYE^{58}NN(T)	1784.6344	(GlcNAc)$_{2}$Man$_{6}$(GlcNAc)$_{2}$	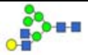
1018.1110(3+)	3052.3171	3052.3098	2	1226.6488	**[NA:140-150]** (L)LNDKHS^{146}NGTIK(D)	1825.6610	Gal$_{2}$(GlcNAc)$_{3}$Man$_{3}$(GlcNAc)$_{2}$	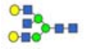
1044.7620(3+)	3132.2703	3132.2666	1	1226.6488	**[NA:140-150]** (L)LNDKHS^{146}NGTIK(D)	1905.6178	Gal$_{2}$(SO$_{3}$)(GlcNAc)$_{3}$Man$_{3}$(GlcNAc)$_{2}$	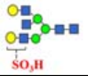
1112.4561(3+)	3335.3526	3335.3460	2	1226.6488	**[NA:140-150]** (L)LNDKHS^{146}NGTIK(D)	2108.6972	Gal$_{2}$(SO$_{3}$)(GlcNAc)$_{4}$Man$_{3}$(GlcNAc)$_{2}$	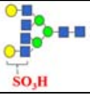
1166.4732(3+)	3497.4039	3497.3988	1	1226.6488	**[NA:140-150]** (L)LNDKHS^{146}NGTIK(D)	2270.7500	Gal$_{3}$(SO$_{3}$)(GlcNAc)$_{4}$Man$_{3}$(GlcNAc)$_{2}$	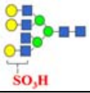
1215.1589(3+)	3643.4610	3643.4567	1	1226.6488	**[NA:140-150]** (L)LNDKHS^{146}NGTIK(D)	2416.8079	Gal$_{3}$(SO$_{3}$)(GlcNAc)$_{4}$Man$_{3}$Fuc(GlcNAc)$_{2}$	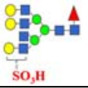
1220.4889(3+)	3659.4510	3659.4516	0	1226.6488	**[NA:140-150]** (L)LNDKHS^{146}NGTIK(D)	2432.8028	Gal$_{4}$(SO$_{3}$)(GlcNAc)$_{4}$Man$_{3}$(GlcNAc)$_{2}$	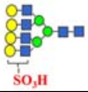
1288.1856(3+)	3862.5411	3862.5310	2	1226.6488	**[NA:140-150]** (L)LNDKHS^{146}NGTIK(D)	2635.8822	Gal$_{4}$(SO$_{3}$)(GlcNAc)$_{5}$Man$_{3}$(GlcNAc)$_{2}$	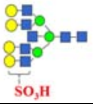
